# Serum Metabolomic Analysis of Coronary Heart Disease Patients with Stable Angina Pectoris Subtyped by Traditional Chinese Medicine Diagnostics Reveals Biomarkers Relevant to Personalized Treatments

**DOI:** 10.3389/fphar.2021.664320

**Published:** 2021-06-14

**Authors:** Na Guo, Peili Wang, Jiaying Yang, Xiaofang Yang, Monique van der Voet, Marjolein Wildwater, Junying Wei, Xuan Tang, Mei Wang, Hongjun Yang

**Affiliations:** ^1^ Experimental Research Center, China Academy of Chinese Medical Sciences, Beijing, China; ^2^ State Key Laboratory Breeding Base of Dao-di Herbs, National Resource Center for Chinese Materia Medica, Center for Post-doctoral Research, China Academy of Chinese Medical Sciences, Beijing, China; ^3^ Xiyuan Hospital, China Academy of Chinese Medical Sciences, Beijing, China; ^4^ College of Pharmacy, Heilongjiang University of Traditional Chinese Medicine, Harbin, China; ^5^ Vivaltes B.V., Heerhugowaard, Netherlands; ^6^ Institute of Chinese Materia Medica, China Academy of Chinese Medical Sciences, Beijing, China; ^7^ School of Chinese Materia Medica, Tianjin University of Traditional Chinese Medicine, Tianjin, China; ^8^ LU-European Center for Chinese Medicine and Natural Compounds, Institute of Biology, Leiden University, Leiden, Netherlands

**Keywords:** angina pectoris, coronary heart disease, traditional Chinese medicine subtypes, metabolomics, vascular endothelial damage

## Abstract

To improve the treatment of patients with coronary heart disease (CHD), personalized treatments based on potential biomarkers could make a difference. To investigate if such potential biomarkers could be found for CHD inhomogeneous, we combined traditional Chinese medicine based diagnosis with untargeted and targeted metabolomics analyses. Shi and Xu patient subtype groups of CHD with angina pectoris were identified. Different metabolites including lipids, fatty acids and amino acids were further analyzed with targeted metabolomics and mapped to disease-related pathways. The long-chain unsaturated lipids ceramides metabolism, bile acid metabolism were differentially affected in the Xu subtype groups. While, Shi-subtype patients seemed to show inflammation, anomalous levels of bioactive phospholipids and antioxidant molecules. Furthermore, variations in the endothelial damage response and energy metabolism found based on ELISA analysis are the key divergence points between different CHD subtypes. The results showed Xu subtype patients might benefit from long-chain unsaturated lipids ceramides as therapeutic targets. Shi subtype patients might benefit more from levels of polyunsaturated fatty acid consumption and treatments that help in restoring energy balance. Metabolic differences can be essential for treatment protocols. Thus, patient group specific differences can serve as important information to refine current treatment approaches in a personalized manner.

## Introduction

Cardiovascular disease is the leading cause of death worldwide, and the mortality rate of coronary heart disease (CHD) is the highest among cardiovascular diseases (Kaur et al., 2020). Many CHD patients have angina pectoris, which might be due to coronary artery obstruction, anemia, abnormal heart rhythms or heart failure. The major cause is the obstruction or spasm of the arteries that supply blood to the heart muscle, a consequence of atherosclerosis, as part of coronary artery disease (Mendis et al., 2011; Iseme et al., 2017). Despite extensive research efforts, which have resulted in improvements in the diagnosis and treatment of angina pectoris in CHD patients, angina pectoris is still associated with very high rates of sudden cardiac death and myocardial infarction (Benjamin et al., 2019). Given the persistence of these high number of incidences and the substantial global health burden due to CHD with angina pectoris, there is a clear need for the development of novel approaches, such as precision medicine, for the treatment of CHD with angina pectoris.

The causes of cardiovascular diseases were found to be quite diverse in recent studies. The genetic heterogeneity of coronary artery disease, for example, was widely studied using candidate gene approaches and genome-wide association studies (GWAS) (Dai et al., 2016; Khera and Kathiresan, 2017a; Khera and Kathiresan, 2017b). Sex and age heterogeneity of patients with cardiovascular diseases have been well established as well (Bots et al., 2017; Haider et al., 2020; Waheed et al., 2020). Other syndromes and their associated comorbidities, including diabetes, cerebrovascular, peripheral artery disease, and some psychology diseases like major depressive disorder and anxiety have been reported to impact CHD outcomes, also influence the different incidences among the population with CHD (Steffens et al., 2002; Hasimu et al., 2007; Kuppuswamy and Gupta, 2009; Carney and Freedland, 2012). Despite those diversions of CHD above, current treatments mostly focus on a standardized method to reduce the risk of adverse cardiovascular events. While it is clear that treatment approaches that take patient differences into account will be more beneficial identification of these approaches is a real challenge.

Currently, there are no widely accepted standards to classify CHD with angina pectoris into subtypes based on phenotypes or chemical potential biomarkers. Metabolomics-based approaches, which combine high-resolution analytical approaches with statistical analyses, have been increasingly employed in clinical researchers, including research on cardiovascular disease (Lam et al., 2017; Ruiz-Canela et al., 2017; van der Laan et al., 2018; Barba et al., 2019). Multiple recent studies have explored the metabolic profiles of CHD patients with stable angina pectoris (DeRatt et al., 2017; Axton et al., 2018; Chunguo et al., 2018; Zhang et al., 2018; Sun et al., 2019; Wang et al., 2019; Zhong et al., 2019). Those studies provided insights into the mechanism of action of some CHD-related drugs. Metabolic profiles of patients within different CHD subtypes have however not been investigated. The development of a method to subtype patients based on metabolic profiles would help to determine which patients are likely to respond to specific medical interventions and allow for the precision medicine-based treatment of these patients, thereby improving therapeutic outcomes.

Using chronobiology approach to discover development of disease by comparing patients with different symptoms at different stages of CHD is a viable strategy to identify potential biomarkers to subtype CHD, this approach is also time consuming and expensive. An alternative strategy could be to use Traditional Chinese medicine (TCM), an experience-based medicinal approach to classify CHD into different subtypes. At present, many practitioners tend to strictly follow the syndrome differentiation theory and proficiently use personal experience to diagnose patients with stable angina CHD in TCM. According to TCM diagnostics, CHD can be divided into two broad categories that consist of the Shi subtype and the Xu subtype (Zhao et al., 2018; Wei et al., 2019). In reality, simple and handy diagnostic methods are practiced in the clinic, differentiating subtypes of CHD according to the main/minor symptoms such as chest pain and chest tightness and some clinical auxiliary examinations including sublingual vein, tongue coating, and pulse manifestation (Cheng et al., 2014).

We conducted untargeted and targeted metabolomics analyses of serum samples from CHD patients of the Shi and Xu subtypes with stable angina pectoris and healthy control individuals for comparison. We identified biological pathways related to lipids, fatty acids, and amino acids that differentiate between the two subtypes of CHD with stable angina pectoris. These findings thus provide scientific evidence of the TCM subtype classification and can help to further classify a population of CHD patients with stable angina pectoralis diagnosed via Western medicine techniques.

In addition, we analysed indexes of vascular endothelial injury in the patients and combined these findings with those of our metabolic analyses to better explore the mechanistic basis of CHD with stable angina pectoris and the differences between its subtypes. As these metabolic differences might influence treatment efficacy in these patients, they can serve as important additional information to refine treatments in a personalized manner and thus improve treatment efficacy.

## Materials and Methods

### Chemicals and Materials

MS-grade methanol, formic acid, acetic acid, and acetonitrile were purchased from Fisher Scientific (Milford, MA, United States). Ultra-pure water (18.2 MΩ) was prepared with a Milli-Q water purification system (Bedford, MA, United States). Histidine, glutamine, and creatinine were purchased from Tokyo Chemical Industry (Tokyo, Japan). Five types of fatty acids were purchased from Larodan (Stockholm Sweden). Internal standards for 11 amino acids were purchased from Cambridge Isotope Laboratories (Andover, MA, United States). Other standards were purchased from Sigma-Aldrich (St. Louis, MO, United States). Detailed information on the standards including compound names, CAS names and sources are provided (Supplementary Table S1).

Phosphatidylethanolamine (PE; 12:0/13:0), lysophosphatidylcholine (LPC; 19:0/0:0), phosphatidylcholine (PC; 19:0/19:0), sphingomyelin (SM; d18:1/12:0), and ceramide (Cer; d18:1/17:0) standards were procured from Avanti Polar Lipids (Alabaster, AL, United States). Triglyceride TAG (15:0/15:0/15:0), free fatty acid (FFA; C19:0), and d3-FFA (C16:0) were purchased from Larodan, and d3-FFA (C18:0) was purchased from Sigma-Aldrich. All other chemicals and reagents used were of analytical grade. The Human ET-1 QuantiGlo ELISA Kit (lot QET00B), Human sICAM-1/CD54 Allele-specific Quantikine ELISA Kit (lot DCD540), and Human sVCAM-1/CD106 Quantikine ELISA Kit (lot DVC00) were purchased from R&D Systems (Minneapolis, MN, United States). An NO assay kit (lot A012-1-2) and a TXB2 assay kit (lot EM0232) were purchased from Nanjing Jiancheng Bioengineering Institute (Nanjing China), and a 6-Keto-PGFlα ELISA kit (lot 515211-96S) was purchased from Cayman Chemical (Ann Arbor, MI, United States). An ATP ELISA kit (lot CEA349Ge) was purchased from Cloud-Clone (Wuhan, China).

### Participants and Selection Criteria

The patients were recruited from Xiyuan Hospital at the China Academy of Chinese Medical Sciences. Sixty CHD patients with stable angina pectoris (aged 48–75 years) were selected and diagnosed by Western medical standard diagnostic methods such as angiography or CT angiography. The patients were documented with >50% of coronary stenosis in at least one major coronary artery. Then, we have established TCM diagnostic and differentiation criteria for Shi syndrome and Xu syndrome. The subjects were diagnosed by two chief doctors, enabling scaling different symptoms into scores. We determined Shi and Xu based on the TCM syndrome scores of the two syndrome types (Supplementary Table S2). The individuals in the control group were free of CHD and angina-related symptoms. The studied participants provided details regarding to their age, sex, cardiac risk factors, and prior cardiac disease and underwent haematological and biochemical examinations. The Ethics Committee of the Medical Experimental Center of the Chinese Academy of Chinese Medical Sciences approved the collection of samples, and written informed consent was obtained from each subject.

### Western Medicine Diagnostic Criteria

Participants in this study were diagnosed with stable angina pectoris and CHD according to the criteria defined by the International Society of Cardiology, the WHO, the clinical nomenclature standardization joint special group report on “Naming and Diagnostic Criteria of Ischemic Heart Disease” (Cardiology, 1981), and the 2013 European Society of Cardiology guidelines for the management of stable coronary artery disease (Montalescot et al., 2013).

### Traditional Chinese Medicine Diagnostic Criteria

TCM-based diagnosis of the Shi and Xu subtypes of CHD was based on the Standard Program for the Diagnosis and Treatment (2014) of Coronary Heart Disease with Angina Pectoris in Chinese Medicine formulated by the State Administration of TCM of China (China and T.S.A.o.T.o, 2014). The diagnostic criteria of both syndromes included main symptom(s) in combination with at least one minor symptom. The main symptoms of Shi are chest pain and chest tightness, and the minor symptoms are fullness chest, hypochondrium and sighing. Nonetheless, the main symptoms of Xu are chest pain and chest tightness, and the minor symptoms are dizziness, insomnia, and dreaminess. At least five points were needed to place the patients in the Shi (TCM diagnostic criteria: main symptom(s) and at least one minor symptom ≥5); at least three points of differentiation were needed to place the patients in the Xu (TCM diagnostic criteria: main symptom(s) and at least one minor symptom ≥3). Moreover, as an alternative approach for subtyping, the two TCM doctors also classified those included patients into two subtypes based on the patient's tongue manifestation, sublingual vein, tongue coating and pulse manifestation status (Supplementary Table S2). Our classification of Shi and Xu based on differentiation criteria was in accordance with the diagnoses by the doctors according to their experience in checking the condition of tongues and pulses of all patients.

### Sample Collection and Laboratory Measurements

Following collection, blood samples were centrifuged for 10 min at 870×*g* at 4°C, and supernatants (serum) were frozen at −80°C for analysis.

Levels of the following serum biochemical parameters were analysed for all study participants: endothelin-1 (ET-1), nitric oxide (NO), 6-keto prostaglandin F1α (6-Keto-PGF 1α), soluble vascular cell adhesion molecule-1(sVCAM-1), soluble intercellular adhesion molecule-1 (sICAM-1), thromboxane B2 (TXB2), and adenosine triphosphate (ATP). ET-1 was analysed by chemiluminescence enzyme immunoassay using a LUMO Chemical Luminescence Immunity Analyzer from Auto Biological Company (Zhengzhou, China). NO was analysed by spectrophotometry using a model 721 spectrophotometer from Yili Fine Chemical Company (Shanghai, China). TXB2, sICAM-1, sVCAM-1, ATP, and 6-Keto-PGF 1α were analysed by ELISA using SpectraMAX M2 multi-mode microplate readers from Molecular Devices Company (Silicon Valley, CA, United States of America). Assays to detect the above five indicators were performed in 96-well ELISA plates. The ELISA kits included ELISA buffer, washing buffer, extraction buffer, substrate, standard enzyme conjugate, and antibody-coated plates. All assays were performed according to the manufacturers’ instructions.

### Standard and Sample Preparation

Standard stock solutions at 1 mg/ml were prepared in methanol or water and stored at 4°C.

Untargeted serum metabolomics analyses were conducted by mixing 50 μL of serum with 150 μL of cold methanol containing the following standards: LPC (19:0/0:0) (37.5 ng/ml), PE (12:0/13:0) (500 ng/ml), SM (d18:1/12:0) (50 ng/ml), and phenylalanine-d5 (100 ng/ml). The mixtures of serum samples and standard solutions were vortexed for 1 min and centrifuged for 10 min at 14,000×*g* at 4°C. The supernatants were immediately analysed via ultra-performance liquid chromatography/ Time of Flight -mass spectrometry (UPLC/TOF-MS) (described below).

For targeted amino acid analyses, 4 µl of serum was mixed with a 991 μL of a solution of 1.7 mm ammonium formate in 85% acetonitrile containing 0.1% formic acid and 5 μL of the each following 11 internal standards: l-alanine-d4 (4.7 ng/ml), l-arginine-13C, d4 (10.8 ng/ml), l-aspartic acid-d3 (6.8 ng/ml), l-citrulline-d2 (8.9 ng/ml), glycine-13C, 15 N (19.3 ng/ml), l-leucine-d3 (6.7 ng/ml), l-methionine-d3 (7.6 ng/ml), l-ornithine-d2 (8.5 ng/ml), l-phenylalanine-13C6 (8.6 ng/ml), l-tyrosine-13C6 (9.4 ng/ml), and l-valine-d8 (6.3 ng/ml). The samples were mixed for 1 min and centrifuged for 5 min at 14,000×*g* at 4°C, after which the supernatants were analysed by ultra-performance liquid chromatography-mass spectrometry/mass spectrometry (UPLC/MS-MS) (described below).

For targeted lipid and fatty acid analyses, 10 μL of serum was mixed with 150 μL of cold methanol supplemented with the following standards: PC (19:0/19:0) (30 ng/ml), LPC19:0 (30 ng/ml), PE (12:0/13:0) (100 ng/ml), Cer (d18:1/17:0) (200 ng/ml), SM (d18:1/12:0) (40 ng/ml), TAG (15:0/15:0/15:0) (200 ng/ml), FFA 16:0-d3 (200 ng/ml), FFA 18:0-d3 (200 ng/ml), and FFA 19:0 (200 ng/ml). The samples were mixed for 30 s, after which 500 µl of MTBE was added, and the samples were agitated at room temperature for 20 min to fully extract the lipids. Next, 125 µl of water was added, and the samples were shaken and centrifuged for 10 min at 16,826×g at 4°C. A 100 µl volume of the sample supernatant was then dried using concentrator and resuspended in 200 µl of a solution of water:isopropanol: acetonitrile [5:30:65 (v/v/v)]. The samples were again shaken for 1 min and centrifuged as described above, and the supernatants were analysed by UPLC/MS-MS (described below).

One-millilitre volumes of each serum sample were pooled to produce a pooled quality control (QC) serum was prepared for each analysis as described above. This sample was used to validate the stability of the LC-MS system (described below).

### UPLC-qTOF-MS

A Xevo G2-XS QTOF-MS (Waters; Micromass MS Technologies, Manchester, United Kingdom) with an electrospray ionization (ESI) source was used in positive and negative ion modes in this study. Separation was achieved with a Waters Acquity UPLC HSS T3 column (100 mm × 2.1 mm, 1.7 μm). The mobile phases used for separation were acetonitrile (A) and water (B); both contained 0.1% (v/v) formic acid. The linear gradient elution settings were as follows: 0–3.5 min, 95–30% B; 3.5–11.0 min, 30–15% B; 11.0–12.5 min, 15–0% B; 12.5–14.0 min, 0%; 14.0–14.1 min, 0–95% B; and 14.1–16.0 min, 95% B. The flow rate was maintained at 0.3 ml/min during separation. The MS settings were as follows: desolvation gas at 800 L/h and 400°C; 50 L/h and 100°C for the cone gas and source temperature, respectively; and 2,000 and 40 V for the capillary and sampling cone, respectively. Mass data were acquired in MS^E^ mode; ramp collision energy at 10–60 V. A LockSpray™ source was used to ensure the reproducibility and accuracy of the MS data collection. The (M + H)+ and (M − H)− ions of leucine-enkephalin were set at *m*/*z* 556.2771 and 554.2615 for the lock mass in positive ESI+ and ESI- modes, respectively. The profiling data for each sample were acquired from 50 to 1,200 Da. Waters MarkerLynx software (Waters; Micromass MS Technologies, Manchester, United Kingdom) was used for data analysis to identify potential biomarkers of CHD patient subgroups. Peak finding, filtering, and alignment were conducted using Waters Progenesis QI Applications Manager (v2.3) (Waters; Micromass MS Technologies, Manchester, United Kingdom). The data collection parameters were as follows: retention time = 0.5–12.5 min; mass = 50–1,200 Da. Automatic mode was used for peak selection, and no specific masses or adducts were excluded. The MetaboAnalyst analytical pipeline (http://www.metaboanalyst.ca/) was used for multivariate statistical analysis of the resultant data (Xia and Wishart, 2011). Partial least squares discrimination analysis (PLS-DA) was used to visualize clustering and trends within the resultant data using MetaboAnalyst analytical pipeline.

### UPLC-MS/MS

Analyses of amino acids, lipids, and fatty acids were conducted using a UPLC system (Waters Acquity) with a quaternary pump, an autosampler, a degasser, and a Xevo TQ-S mass spectrometer with an ESI ionization source. For amino acids, a Waters Acquity UPLC BEH Amide column (2.1 mm × 100 mm, 1.7 μm; flow rate of 0.3 ml/min) was used for separation. The mobile phases for this separation step were 1 mm ammonium formate in acetonitrile containing 0.2% formic acid (A) and 1 mm ammonium formate in water containing 0.1% formic acid (B). The linear elution gradient settings were as follows: 0–0.5 min, 85% A; 0.5–5.5 min, 85–80% A; 5.5–12.5 min, 80–60% A; 12.5–13.0 min 60–85% A, and 13.0–15.0 min, 85% A. The column was warmed to 40°C, and a 5-μl injection volume was used. The column was washed twice between injections with a weak 90% acetonitrile solution and strong 10% acetonitrile solution.

Lipid and fatty acid separation were achieved using a Waters Acquity UPLC BEH C8 column (2.1 × 100 mm, 1.7 μm; flow rate of 0.26 ml/min). The mobile phases for this separation step were 60% acetonitrile containing 5 mm ammonium formate (A) and 90% isopropanol in acetonitrile containing 5 mm ammonium formate (B). The linear elution gradient settings were as follows: 0–1.0 min, 100% A; 1.0–2.0 min, 100–70% A; 2.0–12.0 min, 70–30% A; 12.0–12.5 min, 30–5% A; 12.5–13.0 min, 5–0% A; 13.0–14.0 min, 0% A; 14.0–14.1 min, 0–100% A; and 14.1–16.0 min, 100% A. The column was warmed to 55°C. A 1-μl injection volume was used in positive ion mode, while a 2-μl volume was used in negative ion mode.

Multiple reaction monitoring (MRM) and positive and negative ion modes were used while operating the mass spectrometer (Xevo TQ-S). Data acquisition and peak processing were conducted using Waters TargetLynx software (Waters; Micromass MS Technologies, Manchester, United Kingdom).

### Chromatographic Conditions and MS Method Development

**FIGURE 1:**
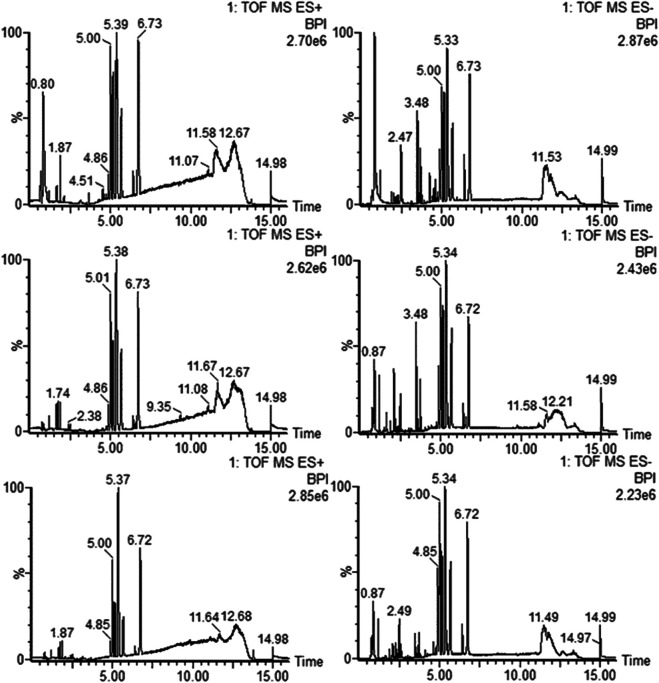
Typical base peak intensity (BPI) chromatograms for serum samples obtained in ESI positive **(A–C)** and negative **(D–F)** modes based on UPLC/TOF-MS: control group **(A, D)**, Shi group **(B, E)**, and Xu group **(C, F)**.

We tested the use of different mobile phases for untargeted metabolomics analysis of the patient serum samples and achieved the best peak resolution using a mixture of acetonitrile (A) and water (B), both containing 0.1% (v/v) formic acid. The data were monitored in positive and negative ion modes for multivariate statistical analysis to collect additional data. The desolvation gas flow rate was set to 800 L/h, while a desolvation temperature of 400°C and flow rate of 0.3 ml/min was used to remove the solvent introduced into the mass spectrometer, and shows representative base peak intensity (BPI) chromatograms for serum collected in negative and positive ESI modes (Figures 1A–F).

For lipid and fatty acid analyses, so we tested a range of injection volumes and extraction times of serum samples under the same conditions to ensure the reliability of the results while minimizing peak overload. After comprehensive consideration, We determined that a 10 μL volume and 20 min extraction period were ideal for achieving the goals of this study. Moreover, before the statistical analysis, we checked the extract ion chromatograms of each compound of each sample to ensure the absence of peak overload. The results of these comparative analyses are presented (Supplementary Figure S1). MRM mode was used for detection. The precursor-to-product ion pair, cone voltage (CV), and collision energy (CE) for each analyte are given (Supplementary Table S3). The details of targeted amino acid analyses are discussed in our previously published study (Guo et al., 2019).

### System Stability

We tested pooled QC samples in positive and negative ion modes to ensure LC-MS system stability throughout the duration of our analyses. These QC samples were processed for analysis in the sample manner as individual samples. The QC samples were injected every 10 injections and analyzed 9 times between samples to verify the stability of the LC-MS system.

### Data Analyses

Data were analysed using our in-house data analytics platform. This data platform contains information from publicly available data sources, omics study data, compound data, and algorithms to mine and connect different factors such as, compounds, text, ontologies, and pathway networks.

ANOVA was used to statistically assess differences in the levels of all tested metabolites obtained by targeted and untargeted analyses, and data were plotted using a histogram with means, standard errors, and 95% confidence intervals. We observed a normal distribution for these analytes.

## Results

### Participant Coronary Heart Disease Diagnosis and Subtyping

In this study, we could subdivide CHD patients diagnosed by conventional Western medicine technologies into two metabolically distinct subgroups using the diagnostic principles of TCM. A total of 90 participants were enrolled in this study: 30 controls, and 60 patients diagnosed with CHD and angina pectoris. The 60 CHD patients were further separated into two equally sized (*n* = 30) subtype groups containing patients with either Xu or Shi subtypes based on TCM, which were diagnosed by two chief Chinese medicine physicians based on main and minor symptoms. Meanwhile, patients with diabetes, poor blood pressure control, severe chronic heart failure, severe arrhythmia, implanted pacemakers, or a history of myocardial infarction and those with liver, renal, haemorrhagic, autoimmune, haematological or mental disorders were excluded from the study group. The study participants provided details regarding their age, sex, cardiac risk factors, and prior cardiac disease and underwent haematological and biochemical examinations. The participant characteristics according to western clinical patrameters were shown in Table 1. No significant differences in age or sex of participants were found among the three groups. The urinary metabolites between male and female participants showed no significant difference (Supplementary Figure S2). On the other hand, clear difference between three groups (control, Xu and Shi subtypes) were observed according to TCM diagnose.

**TABLE 1:** Participants included in the control, Shi-subtype and Xu-subtype groups.

Group	Control group (*n*)		Shi group (*n*)		Xu group (*n*)	
Sex	Male (17)	Female (13)	Male (16)	Female (14)	Male (16)	Female (14)
Age (year)	61.2 ± 1.3	59.2 ± 1.4	59.6 ± 2.0	64.5 ± 1.6	63.0 ± 2.0	63.7 ± 1.8
Average	60.3 ± 1.0		61.9 ± 1.4		63.3 ± 1.3	
Age (year)						
SBP (mmHg)	138.10 ± 4.99		134.60 ± 3.02		129.70 ± 3.04	
DBP (mmHg)	80.84 ± 3.56		81.32 ± 1.70		78.21 ± 2.02	
CR (µmol/L)	79.32 ± 3.41		71.43 ± 2.56		74.74 ± 8.07	
TC (mmol/L)	4.47 ± 0.16		4.13 ± 0.16		4.25 ± 0.24	
TG (mmol/L)	1.10 ± 0.10		1.55 ± 0.20		1.27 ± 0.13	
HDL (mmol/L)	1.37 ± 0.06		1.21 ± 0.06		1.20 ± 0.06	
LDL (mmol/L)	2.21 ± 0.14		2.37 ± 0.14		2.53 ± 0.21	

### Validation of LC-MS Methods

Validation of the LC-MS methods is shown by the overlapping base peak intensity (BPI) chromatograms for these QC samples (Supplementary Figures S3A–SB). The repeatability of the spiked internal standards was used to assess the overall technical variation (OTA) in our untargeted metabolomics analyses (Supplementary Table S4). The low relative standard deviations of retention time and peak areas indicated that the OTA was low and the reproducibility of these measurements over the experimental duration was acceptable. For targeted lipid, fatty acid and amino acid analyses, the majority of the coefficient of variation values corresponding to the internal standard peak area were <30% (Supplementary Tables S5,S6).

### Differences Between the Metabolic Profiles of Healthy Subjects and Patients with Two Coronary Heart Disease Subtypes

**FIGURE 2:**
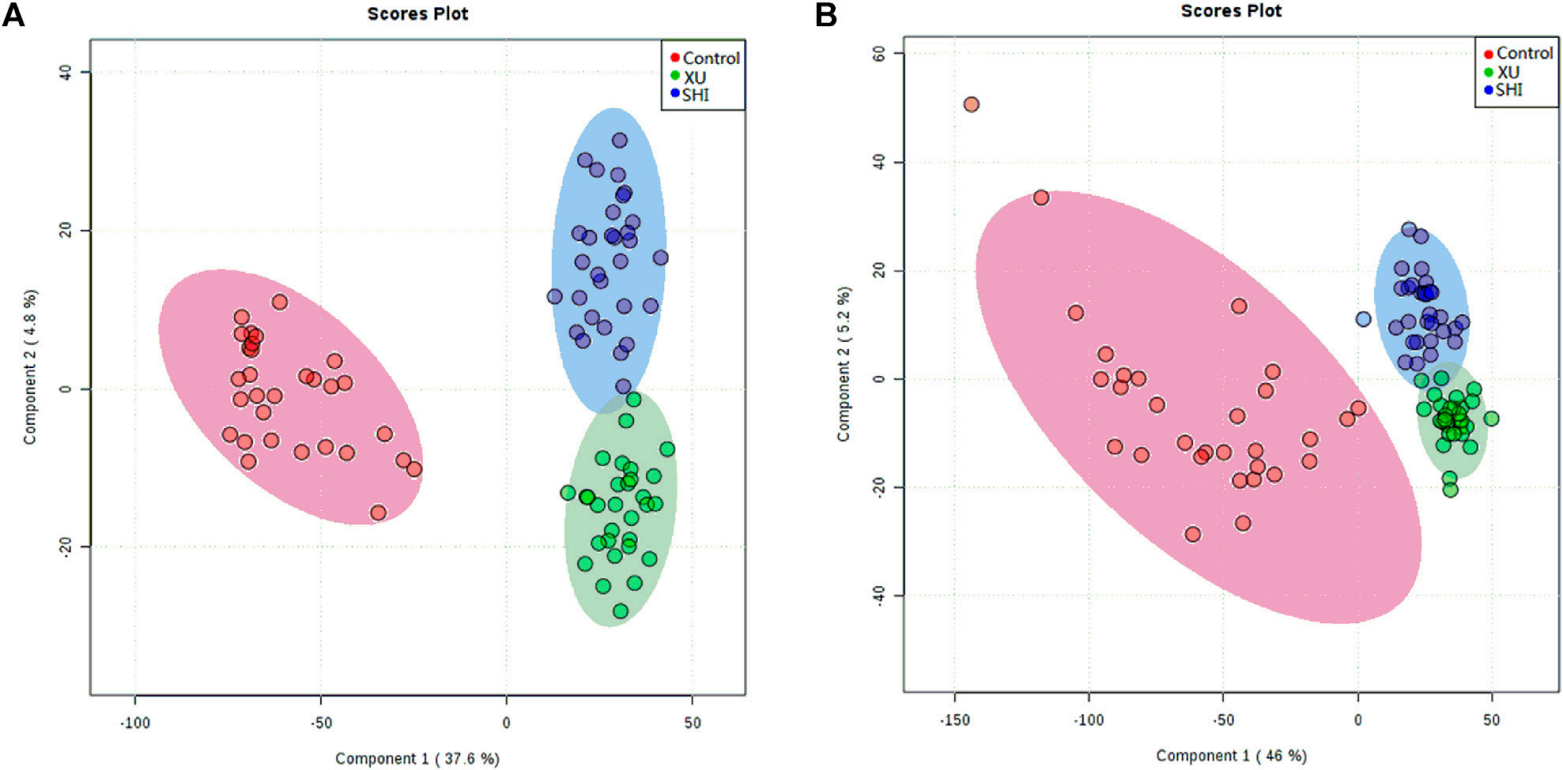
PLS-DA score plots showing control samples (red dots, *n* = 30), Shi-subtype samples (blue dots, *n* = 30) and Xu-subtype samples (green dots, *n* = 30) in ESI positive **(A)** and ESI negative modes **(B)**.

To establish untargeted and targeted metabolomics approaches to compare the serum metabolic profiles of the three groups, we used PLS-DA to analyse the data obtained in both ion modes (Figures 2A,B). We obtained a satisfactory classification indicating that CHD patients with stable angina pectoris including Shi and Xu subtypes exhibited metabolic states that were significantly different from those of the control patients. In addition, we observed separation between CHD patients with the Xu and Shi subtypes, indicating that the serum metabolic profiles of patients with these two TCM-based subtypes of CHD with stable angina pectoris differed. To validate the model, a permutation test was performed (*n* = 200), and the values of *R*2 and *Q*2 (Supplementary Figures S4A,B) indicate that the model showed great fitness and prediction efficacy. Together, these results show that good separation was achieved between these three groups. This observation indicated that CHD patients with the Xu and Shi subtypes disturbed the metabolism of endogenous substances and differently altered the serum metabolic fingerprint in CHD with stable angina pectoris compared to that under control conditions.

### Potential Biomarker Identification and Analysis by Non-targeted and Targeted Metabolomics

To find the potential biomarkers, we imported the LC-MS raw data into Progenesis QI software, more than ten thousands of features were detected in the untargeted metabolomics study. We next identified the metabolites in our analysed samples. Initially, MassLynx software was used to determine the elemental composition of the identified compounds, after which databases including the Kyoto Encyclopaedia of Genes and Genomes (KEGG) (http://www.genome.jp) and HMDB (http://www.hmdb.ca) databases were searched to identify the structures of these potential biomarkers based upon high-resolution MS and MS/MS spectra data. The identities of these metabolites were further confirmed after comparing them to defined standards on the basis of retention time and MS/MS fragmentation patterns (Supplementary Table S7).

**FIGURE 3:**
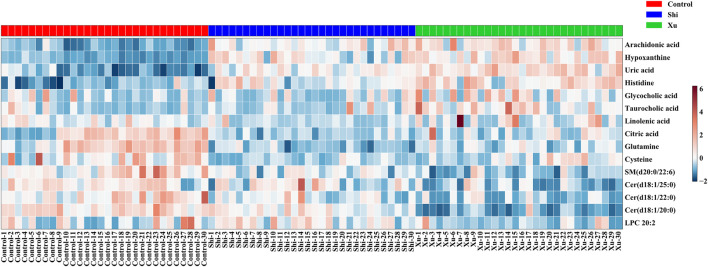
Metabolites whose levels were significantly different between groups were organized into a heat map, with red and blue corresponding to increased and decreased levels, respectively. There are seven metabolites, i.e., Linolenic acid, Citric acid, Uric acid, Taurocholic acid, Hypoxanthine, Arachidonic acid, and Glycocholic acid coming from the data set of unbiased analysis using UPLC-qTOF-MS, the nontargeted metabolomics study. There were eight metabolites, i.e., Histidine, Cysteine, Glutamine, LPC (20:2), Cer (d18:1/20:0), Cer (d18:2/22:0), Cer (d18:1/25:0), and SM (d20:0/22:6) coming from the data set of the targeted metabolomics study using QqQ-MS.

**FIGURE 4:**
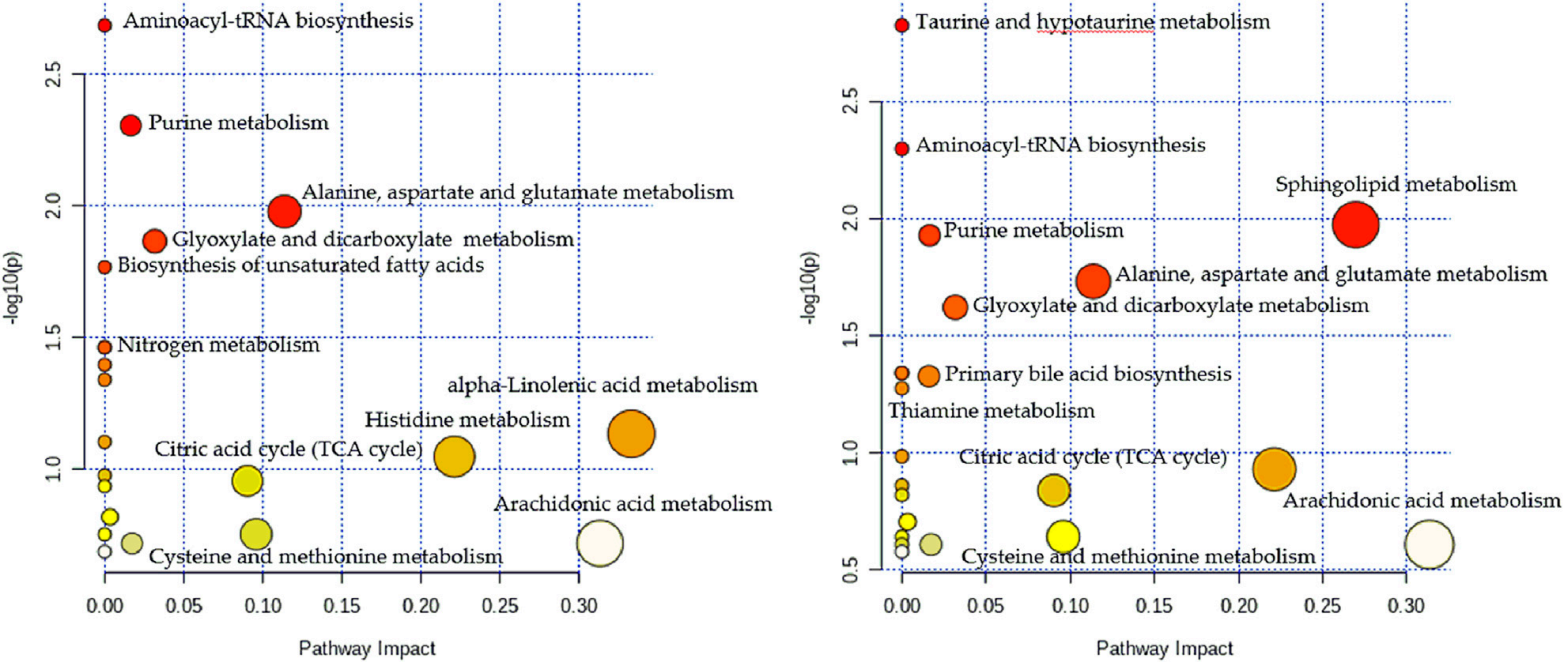
Metabolomics pathway analysis indicating select metabolic pathways that are significantly affected in the Shi **(A)** and Xu **(B)** subtypes of CHI). *p* values are shown on the *Y*-axis, and pathway impact values are shown on the *X*-axis. The nodes are coloured based on *p*-value, while node sizes indicate impact values.

To gain further insights into the hydrophilic and hydrophobic metabolite profiles after non-targeted analyses, we conducted targeted analyses of serum amino acids, lipids, and fatty acids. While in the targeted lipid, fatty acid, and amino acid metabolomics experiments, there were 141 lipids, 23 fatty acids, and 22 amino acids detected, respectively. To identify the metabolic potential biomarkers specific for the two CHD subtypes, we used independent samples t-tests to compare the data, using *p* < 0.05 as the significance threshold. Significant differences in the abundance of these metabolites were further confirmed in Table 2. As shown in Table 2, when comparing the control and the Shi CHD subtype, levels of the long-chain unsaturated lipids ceramides (Cers) and sphingomyelins were significantly lower in the Xu CHD subtype, while levels of glycocholic acid, taurocholic acid, arachidonic acid, histidine, uric acid and hypoxanthine were higher in the Xu CHD subtype. Shi-subtype CHD patients show increased levels of LPC (20:2) and downregulated levels of citric acid, linolenic acid, cysteine and glutamine, compared with the control and the Xu CHD subtype. We organized the putative metabolic potential biomarkers into a heat map to show their distribution (Figure 3). We next explored the potential pathways involved in regulating the differentially abundant metabolites detected in the above analysis using MetaboAnalyst, with those pathways that had low *p*-values and high pathway impacts (Figures 4A,B). Metabolites in Shi-subtype CHD patients were primarily associated with the following metabolic pathways (Figure 4A): Aminoacyl-tRNA biosynthesis, Purine metabolism, Alanine/aspartate and glutamate metabolism, Glyoxylate and dicarboxylate metabolism, Cysteine and methionine metabolism. In addition, 21 metabolic pathways were identified (Supplementary Table S8). Metabolites in Xu CHD subtype patients were primarily associated with the following metabolic pathways (Figure 4B): Taurine and hypotaurine metabolism, Aminoacyl-tRNA biosynthesis, Sphingolipid metabolism, Purine metabolism, Alanine/aspartate and glutamate metabolism, Glyoxylate and dicarboxylate metabolism. In addition, 22 metabolic pathways were identified when comparing control subjects. For full details of affected pathways (Supplementary Table S9). Detailed metabolite differences between Xu and Shi subtype CHD patients were shown (Supplementary Table S10).

**TABLE 2:** Identification of potential biomarkers in serum.

No	Identification	Fold change (trend)			FDR			Pathway
	Results							
		Control VS Shi	Control VS Xu	Shi VS Xu	Control VS Shi	Control VS Xu	Shi VS Xu	
1	Histidine	1.447(↑)	1.689(↑)	1.167(↑)	0.000	0.000	0.019	Aminoacyl-tRNA biosynthesis; histidine metabolism; beta-Alanine metabolism
2	Cysteine	0.563(↓)	0.743(↓)	1.321(↑)	0.000	0.034	0.014	Aminoacyl-tRNA biosynthesis; Taurine and hypotaurine metabolism; Cysteine and methionine metabolism; Glycine, serine and threonine metabolism; Thiamine metabolism; Pantothenate and CoA biosynthesis; Glutathione metabolism
3	Glutamine	0.706(↓)	0.761(↓)	1.077(↑)	0.000	0.000	0.038	Glyoxylate and dicarboxylate metabolism; Alanine, aspartate and glutamate metabolism; D-Glutamine and D-glutamate metabolism; Nitrogen metabolism; Arginine biosynthesis; Aminoacyl-tRNA biosynthesis; Purine metabolism; Pyrimidine metabolism
4	Uric acid	1.681(↑)	2.037(↑)	1.212(↑)	0.000	0.000	0.000	Purine metabolism
5	Hypoxanthine	4.193(↑)	5.933(↑)	1.415(↑)	0.000	0.000	0.005	Purine metabolism
6	Arachidonic acid	2.299(↑)	2.735(↑)	1.19(↑)	0.000	0.000	0.049	Biosynthesis of unsaturated fatty acids; Arachidonic acid metabolism
7	Linolenic acid	0.593(↓)	1.518(–)	2.559(↑)	0.000	0.067	0.018	Biosynthesis of unsaturated fatty acids; alpha-Linolenic acid metabolism
8	Citric acid	0.667(↓)	0.83(–)	1.244(↑)	0.049	0.221	0.028	Tricarboxylic acid cycle (TCA cycle); Glyoxylate and dicarboxylate metabolism; Alanine, aspartate and glutamate metabolism
9	LPC (20:2)	1.099(↑)	0.641(–)	0.583(↓)	0.048	0.487	0.006	Glycerophospholipid metabolism
10	Taurocholic acid	1.334(–)	2.639(↑)	1.979(↑)	0.396	0.000	0.009	Taurine and hypotaurine metabolism; Primary bile acid biosynthesis
11	Glycocholic acid	1.008(–)	1.941(↑)	1.925(↑)	0.981	0.022	0.019	Primary bile acid biosynthesis
12	SM(d20:0/22:6)	0.732(–)	0.576(↓)	0.788(↓)	0.888	0.008	0.006	Sphingolipid metabolism
13	Cer(d18:1/20:0)	0.761(–)	0.272(↓)	0.358(↓)	0.892	0.000	0.000	Sphingolipid metabolism
14	Cer(d18:2/22:0)	0.599(–)	0.334(↓)	0.558(↓)	0.279	0.000	0.015	Sphingolipid metabolism
15	Cer(d18:1/25:0)	0.874(–)	0.477(↓)	0.546(↓)	0.342	0.002	0.006	Sphingolipid metabolism

FDR: *p*-value corrected by false discovery rate; “↑” means a higher level of metabolites; “↓” means a lower level of metabolites; “–” represents no statistically significant difference Control represents control group; Shi represents Shi subtype group; Xu represents Xu subtype group.

### The Relevance of Damage to the Vascular Endothelium and Metabolic Imbalance

**FIGURE 5:**
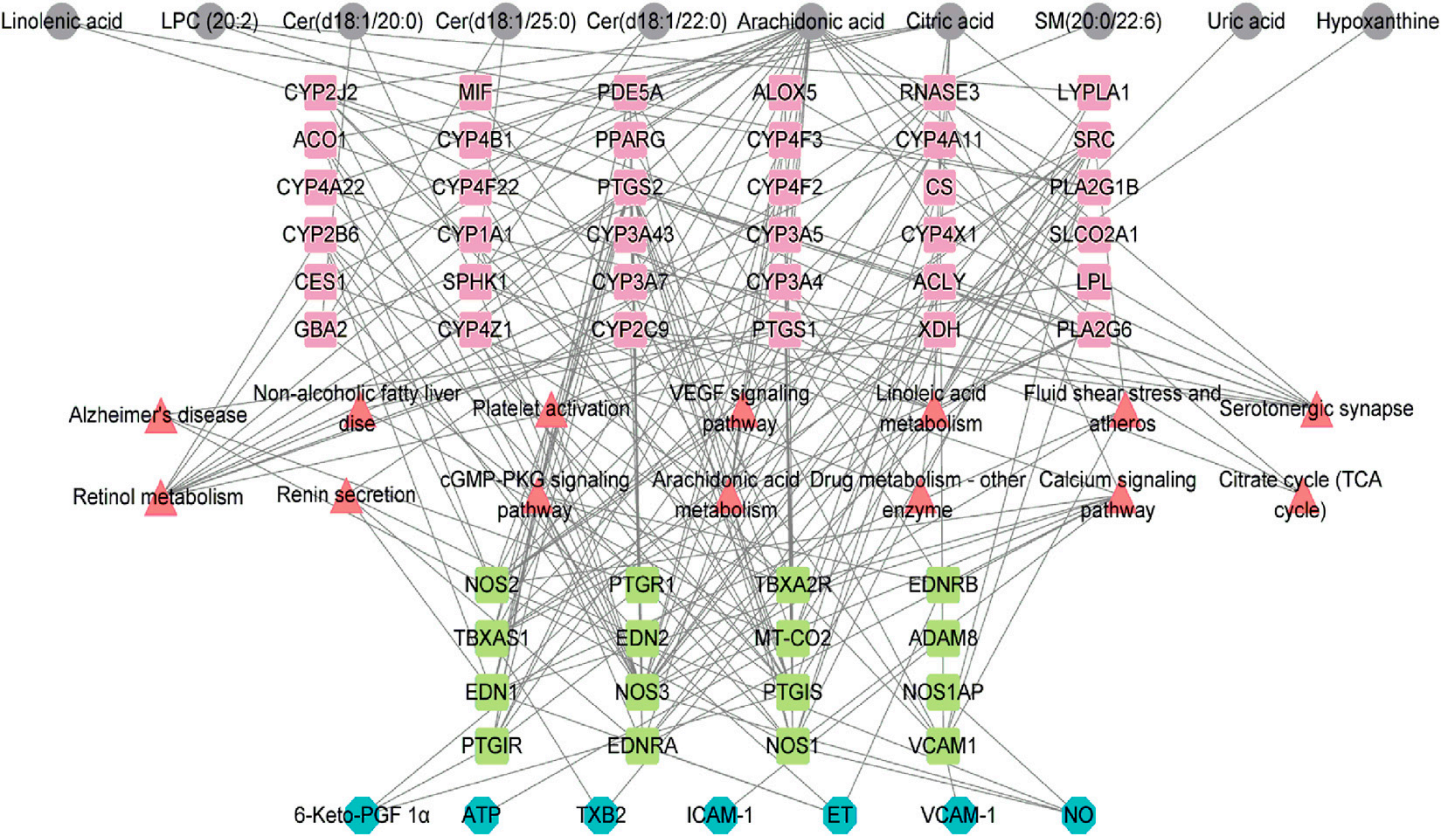
Network showing the interactions among indexes related to vascular endothelial damage, ATP, protein targets, metabolic pathways that arc significantly altcrcd in CHD, and the identified metabolites. Grey nodes represent the identified metabolites; red nodes represent pathways related to these metabolites; pink and grccn nodes represent the identified protein targets; bluc nodes rcprcscnt thc relatcd indcxcs of vascular cndothclial damagc and ATP.

**FIGURE 6:**
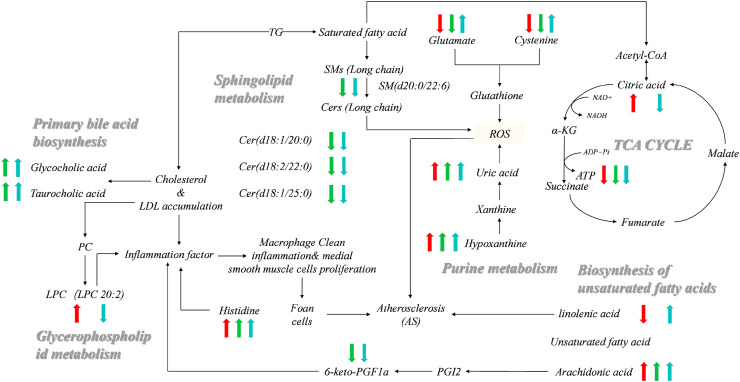
Networks of potential serum biomarkers of CHD that varied among the healthy control, Shi-subtype, and Xu-subtype groups. Arrows (“↑↓”) indicated the significantly upregulated or downregulated metabolites in the Shi (red) and Xu (green) patients compared to the healthy control, respectively. The significant changes of metabolites in the Shi group compared to the Xu group are also indicated (blue). There are seven metabolites, i.e., Linolenic acid, Citric acid, Uric acid, Taurocholic acid, Hypoxanthine, Arachidonic acid, and Glycocholic acid coming from the data set of unbiased analysis using UPLC-qTOF-MS, the non-targeted metabolomics study. There were eight metabolites, i.e., Histidine, Cysteine, Glutamine, LPC (20:2), Cer (d18:1/20:0), Cer (d18:1/22:0), Cer (d18:1/25:0), and SM (d22:0/22:6) coming from the data set of the targeted metabolomics study using QqQ-MS.

The metabolite-related protein interaction network was constructed through the STRING database (minimum required interaction score >0.4). A regulatory network containing these indentified pathways, proteins, and significant molecular metabolites (Figure 5) was constructed using Cytoscape 3.8.2. Significant interactions between 6-Keto-PGFlα, NO, ET-1, sICAM-1, sVCAM-1, TXB2, ATP, and identified significant molecular metabolites, metabolic pathways were examined. To investigate how vascular endothelial function and energy metabolism were affected in the different CHD subtypes, a set of endothelial markers such as 6-Keto-PGFlα, NO, ET, sICAM-1, sVCAM-1, TXB2, and energy marker, ATP were examined. We observed significant reductions in 6-Keto-PGFIα and ATP levels in CHD patients relative to controls (Table 3); ET-1 levels were significantly higher in CHD patients as compared to those in control. Although sICAM-1 and sVCAM-1 levels were not significantly different from control levels in patients with Shi-type CHD, there was a significant increase in these sICAM-1, sVCAM-1, and ET-1 levels in Xu-type CHD patients relative to those in patients with Shi-type disease. In addition, ATP and 6-Keto-PGFIα levels were significantly lower in patients with Shi-type disease as compared with patients with Xu-type CHD. These results thus suggest that vascular endothelial function is impaired in CHD patients with stable angina and that these impairments are more severe in patients with Xu-type CHD as compared with patients with Shi-type CHD. All the differences between XU and SHI subtypes were mapped. We identified alterations in three metabolic differences that were characteristic for the Xu subtype: the long-chain unsaturated lipids Cers and sphingomyelins metabolism, bile acid metabolism and vascular endothelial dysfunction. For the Shi subtype, we also could identify a Shi type specific CHD fingerprint, Shi patients showed inflammation, increased bioactive phospholipids and disrupted energy metabolism pathways (Figure 6).

**TABLE 3:** Biochemical indexes of participants in the control, Shi-subtype and Xu-subtype groups.

Index	Control (*n* = 30)	Shi (*n* = 30)	Xu (*n* = 30)
ET-1 (pg/ml)	1.08 ± 0.09	1.73 ± 0.08 (#)	2.16 ± 0.19 (#△)
6-Keto-PGFlα (pg/ml)	604.50 ± 34.78	615.20 ± 31.23	502.40 ± 32.76 (#△)
sICAM-1 (ng/ml)	242.50 ± 9.53	258.00 ± 13.09	322.50 ± 23.02 (#△)
sVCAM-1 (ng/ml)	840.70 ± 32.88	785.50 ± 23.02	934.60 ± 53.56 (△)
ATP (ng/ml)	222.80 ± 9.98	185.00 ± 10.85 (#)	145.70 ± 7.29 (#△)
NO (µmol/L)	23.17 ± 1.08	15.43 ± 1.59 (#)	15.05 ± 1.34 (#)
TXB2 (pg/ml)	75.24 ± 6.29	107.00 ± 4.80 (#)	103.60 ± 4.39 (#)

“#” and “△” indicate *p* < 0.05, independent t-test; “#”, compared to the control group; “△”, compared to the Shi-subtype group.

## Discussion

Reducing the risk of adverse cardiovascular events is crucial for the management of patients with CHD. Since the symptoms of CHD are diverse (Wang et al., 2010), a more personalized treatment approach than currently available methods would benefit patients. Western medicinal approach is targeted and with molecular understanding whilst Chinese medicinal approach is to observe the overall performance of the human body based on experience without molecular evidence. A combination of Western medicine technology with TCM diagnostic knowledge could be an optimal approach to identify personalized molecular markers for different subtypes of patients. We found that TCM subtyping reflected subtype-specific differential metabolite profiles which can be linked to the appearance and/or occurrence CHD related symptoms.

Our finding are that three important and characteristic metabolic patterns define the Xu subtype of CHD. The first specific metabolic characteristic specific to the Xu subtype is downregulation of the long-chain unsaturated lipids Cers and sphingomyelins. Cer metabolites are vital as signaling intermediates in vascular homeostasis, inflammation, stress, apoptosis, and autophagic responses (Xiaoyang, 2019; Tomczyk and Dolinsky, 2020). It has been reported that Cer containing long-chain unsaturated fatty acids (>20 carbons) can shield cells from oxidative stress-induced apoptosis; the reduced availability of Cers therefore found to increase fibrosis, endoplasmic reticulum stress, and apoptosis in heart cells (Lemaitre et al., 2019). In this study, all of the three individually analysed Cer molecules containing long-chain unsaturated acids [Cer (d18:1/20:0), Cer (d18:2/22:0), and Cer (d18:1/25:0)] were downregulated in patients with the Xu subtype of CHD. This finding is particularly exciting as Cer has recently begun to draw attention due to its therapeutic potency. Cers not only exert antidiabetic and antitumor activity but also can act as a potential therapeutic target for atherosclerosis (AS) and can reverse multidrug resistance (Li and Chiang, 2009; Abdelhamid et al., 2018). Therefore, it might be beneficial to investigate the potential role of long-chain unsaturated lipids Cers as therapeutic targets for the Xu subtype of CHD, and that can be further explored to guide personalized intervention strategies.

In addition to the finding that downregulated long-chain unsaturated lipids is characteristic of the Xu subtype of CHD, we also found significantly higher levels of glycocholic acid and taurocholic acid, cholesterol-derived bile acids, in the liver (Russell and Setchell, 1992; Li and Chiang, 2009). Bile acids are biological detergents and metabolic regulators required for proper lipid digestion and absorption. In addition, bile acids facilitate the excretion of cholesterol and other molecules, such as xenobiotics (Batta et al., 1999). Multiple studies have reported a negative correlation between CHD and bile acid excretion, which is also confirmed in our study (Assmann et al., 2006; Gylling et al., 2009). Bile acid accumulation in the blood can directly drive vascular endothelial cell dysfunction and inflammation, resulting in vascular endothelial damage, consistent with our results regarding endothelial indexes (Qin et al., 2006). Bile acid metabolic disorder can also affect lipid metabolism, resulting in hyperlipidemia, which further favours CHD development (Yanwei Gong 2015). After observing differential patterns of bile acid derivative levels in the sera of patients with Xu-subtype and Shi-subtype CHD, we hypothesize that the increases in these levels in patients with Xu-subtype CHD compared to those with Shi-subtype CHD are the results of reduced bile acid excretion. Therefore, based on this hypothesis, lipid-lowering statin drugs (e.g., rosuvastatin, lovastatin, gemfibrozil, simvastatin, and pravastatin), which reduce cholesterol production in the liver (Robinson, 2008), might benefit patients with Xu-subtype CHD as these drugs might attenuate bile acid accumulation as they block cholesterol synthesis.

A third feature characteristic of the Xu subtype of CHD is the disruption of vascular endothelial function and inflammation. Patients with Xu-subtype CHD showed a specific and significant decrease in some parameters reported to be the indicators for the damage of vascular endothelial such as 6-keto-PGF1α, a powerful vasodilator that inhibits platelet aggregation (Rémy Jouve et al., 1984). Studies have shown that CHD and myocardial infarction in patients with endothelium and other factors due to long-term ischemia, hypoxia and endothelium chronic injury, the release of platelet activating factor (PAF) and other inflammatory substances, to decrease the release of 6-keto-PGF1α (Qinhua Chen, 2013). Furthermore, ATP is the most direct source of energy in organisms, during ischemic reperfusion stop the ATP synthesis, increase hypoxanthine, thereby generating reactive oxygen species (ROS) at the time of reperfusion (Kumbasar et al., 2014). Hypoxanthine further oxidized into uric acid (Shao et al., 2017). Uric acid can lead to impaired vascular endothelial function (Feig et al., 2008), it also can increase ROS products and promote oxidative modification of low-density lipoprotein (LDL), oxidized LDL can form foam cells after being swallowed by macrophages, which is an important pathogenesis of atherosclerosis (Zhang et al., 2014). At the same time, studies have shown that the vasoconstrictor ET-1 also may induce subepicardial ischemia and significantly enhance the release of myocardial purine metabolites (Haynes and Webb, 1994). ET-1 is antagonistic bioactive factors, which play an important role in the regulation of cardiovascular system function. Participate in a variety of pathological processes of cardiovascular disease. The injury of vascular endothelial cells increased the release of ET-1 and led to vasoconstriction (Zima et al., 2002). We are concerned that the levels of uric acid, hypoxanthine and ET-1 are higher in Xu-subtype CHD than those in control group and Shi subtype group, and ATP is significantly reduced in the Xu-subtype CHD, which means that in addition to aggravating vascular endothelial damage, also promote the formation of foam cells, which is more likely to lead to cardiovascular disease. In addition, the levels of arachidonic acid (AA), which in addition to its platelet aggregative function, has a pro-inflammatory function, were enhanced in patients with Xu-subtype CHD compared to Shi-subtype (Halade et al., 2012; Chang et al., 2016). The presence of significantly higher levels of amino acids, such as histidine, in the Xu subtype of CHD further supports enhanced inflammation levels and endothelial damage. Interestingly, 6-keto-PGF1α sodium salt has been used to treat pulmonary hypertension (Li et al., 1994) and might thereby provide a simple measure to attenuate the disruption of vascular endothelial function induced by reduced levels of 6-keto-PGF1α in Xu-subtype CHD patients, and drugs such as allopurinol and benzbromarone that can lower the organism’s uric acid levels may be beneficial for Xu-subtype CHD.

Similarly, Shi-subtype CHD patients seemed to show increased levels of inflammation, which may have occurred through different mechanisms. One characteristic specific to patients with Shi-subtype CHD is a decrease in the levels of linolenic acid, an omega-3 polyunsaturated fatty acid (PUFA) that has been shown to lower circulating lipid levels, reduce inflammation, and stabilize AS plaques (Zehr and Walker, 2018). The results of a previous study investigating the effects of PUFA consumption on cardiovascular disease were unclear (Assmann et al., 2006). Although PUFA intake caused a mild reduction in CHD and cardiovascular disease events and slightly reduced the risk of CHD-related death and stroke events, the effects on other major cardiac and cerebrovascular events and related deaths were unclear or not examined (Assmann et al., 2006). Notably, the differences in inflammation between the Shi and Xu subtypes of CHD observed in the current study suggest that PUFA intake might differentially impact patients with these CHD subtypes. Therefore, future studies on the effect of PUFA on CHD should consider different CHD subclasses, including different TCM subtypes.

The second characteristic of patients with the Shi subgroup of CHD is that the levels of bioactive phospholipids were increased. Elevated levels of bioactive phospholipids, such as LPC (20:2), in CHD patients, LPCs may be positively associated with risks of CVD outcomes (Ding and Rexrode, 2020). It is consistent with the previous work of. (Matsumoto et al., 2007). Bioactive phospholipids play diverse roles in cell metabolism and pathological mechanisms. Further studies are expected to confirm how the elevated levels of these lipids affect the progression of the Shi subtype of CHD.

Finally, we observed decreased levels of several core intermediates of the tricarboxylic acid (TCA) cycle, antioxidant molecules (cysteine and glutamine) in the Shi-subtype group compared to the Xu-subtype group. Therefore, drugs such as trimetazidine and ranolazine (drugs to reduce angina symptoms) might be beneficial for patients with Shi-subtype CHD because these drugs can restore energy homeostasis and general health in patients.

In the present study, the personalized diagnosis of TCM can help distinguish patients of stable angina pectoris with different subtypes. We established untargeted and targeted metabolomics approaches to compare the serum metabolic profiles of participants, and identified potential biomarkers that are able to distinguish CHD patients into two subgroups. Compared with those existing clinical chemistry readouts and biochemical indexes (Tables 1, 3), metabolomics aims at a comprehensive characterization of the total metabolome in a biological system (Schiffer et al., 2019). The metabolic profiling *in vivo* based on metabolomics research could provide an integrated view of whole body metabolic disturbances in different disease states (McGarrah et al., 2018). The results implicated that the combination of metabolomics and TCM diagnosis can reveal metabolic characteristics of diseases, indicating that metabolomics is a powerful tool in personalized medicine (Azad and Shulaev, 2018). Our results show that each of these subgroups has characteristic CHD related metabolic pathway defects that might be targeted with subgroup specific therapeutic intervention. For example, Xu subtype patients might benefit from long-chain unsaturated lipids Cers as therapeutic targets and lipid-lowering statins, allopurinol and benzbromarone that can lower the level of uric acid. Nevertheless, Shi subtype patients might benefit more from levels of polyunsaturated fatty acid consumption as well as treatments that help in restoring energy balance. Our study is the first step towards the personalized treatment. The validation of potential markers in independent cohorts with larger sample sizes from different areas (multi-center) and the validation oftherapeutic interventions in different subtype groups of CHD patients are essential and will be next steps of near further studies in our research group.

## Conclusion

In conclusion, the results of our study suggest that the metabolic profile of CHD differs among patients with different subtypes. Variations in the endothelial damage response and lipid and energy metabolism act as key divergence points between these different CHD subtypes, suggesting the potential of targeting of these processes in the development of personalized treatments for patients with CHD and angina pectoralis. Our results show that subtyping patients using TCM can reveal quantifiable differences in metabolism that can then be used to refine current treatment approaches. Integration of the biological knowledge of TCM-driven CHD patient subgroups by TCM experts, analytical chemists, and data scientists might facilitate refined patient group-derived treatment protocols and connect Western diagnose to TCM-based diagnosis. The presented technique (subgrouping by TCM diagnosis for combination with Western methods) could also be used for other diseases.

## Data Availability

The original contributions presented in the study are included in the article/Supplementary Material, further inquiries can be directed to the corresponding authors.
