# Supplementary material for: Serum Metabolomic Analysis of Coronary Heart Disease Patients with Stable Angina Pectoris Subtyped by Traditional Chinese Medicine Diagnostics Reveals Biomarkers Relevant to Personalized Treatments
Source: Front Pharmacol. 2021 Jun 14;12:664320. doi: 10.3389/fphar.2021.664320 (PMC8236985; doi:10.3389/fphar.2021.664320)
Supplement: Supplementary file 1 [file DataSheet1.PDF]

Supplementary Material

1.1 Supplementary Figures

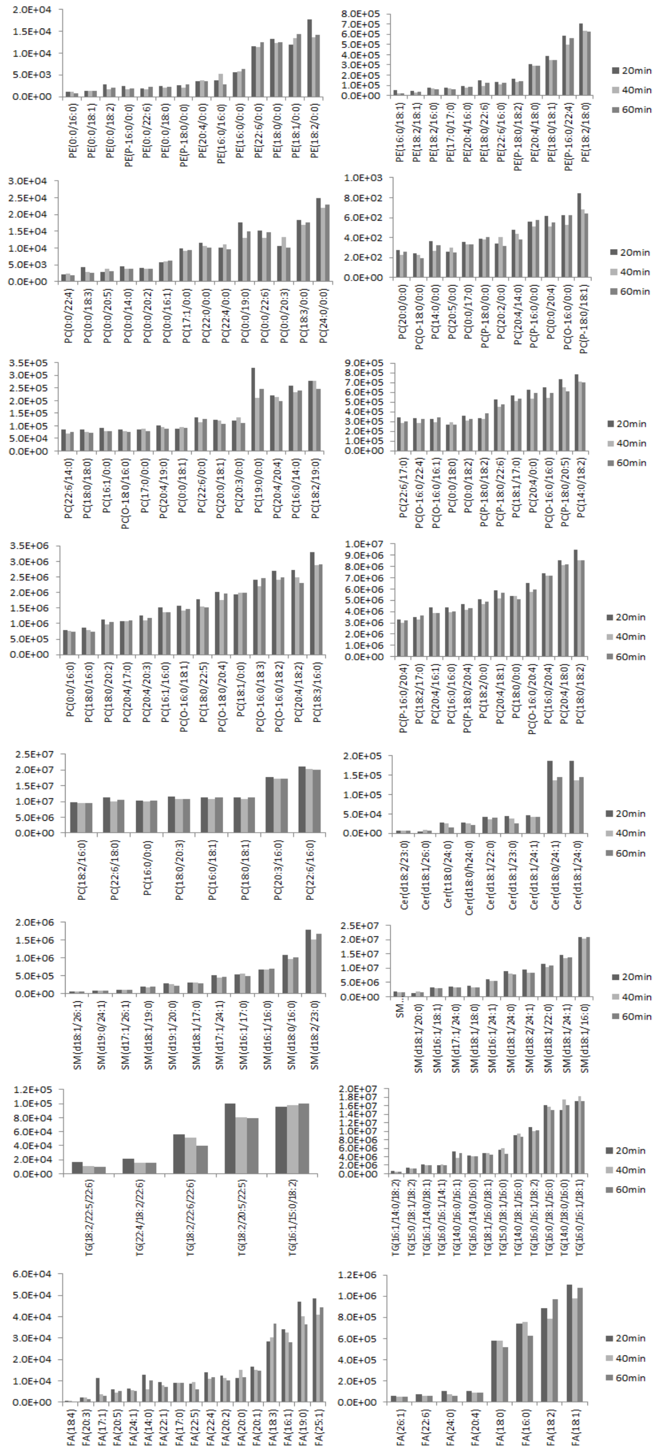

Supplementary Figure 1. Bar chart of all targeted lipids and fatty acids in the serum extracted in shaking time (20 min, 40 min and 60 min).

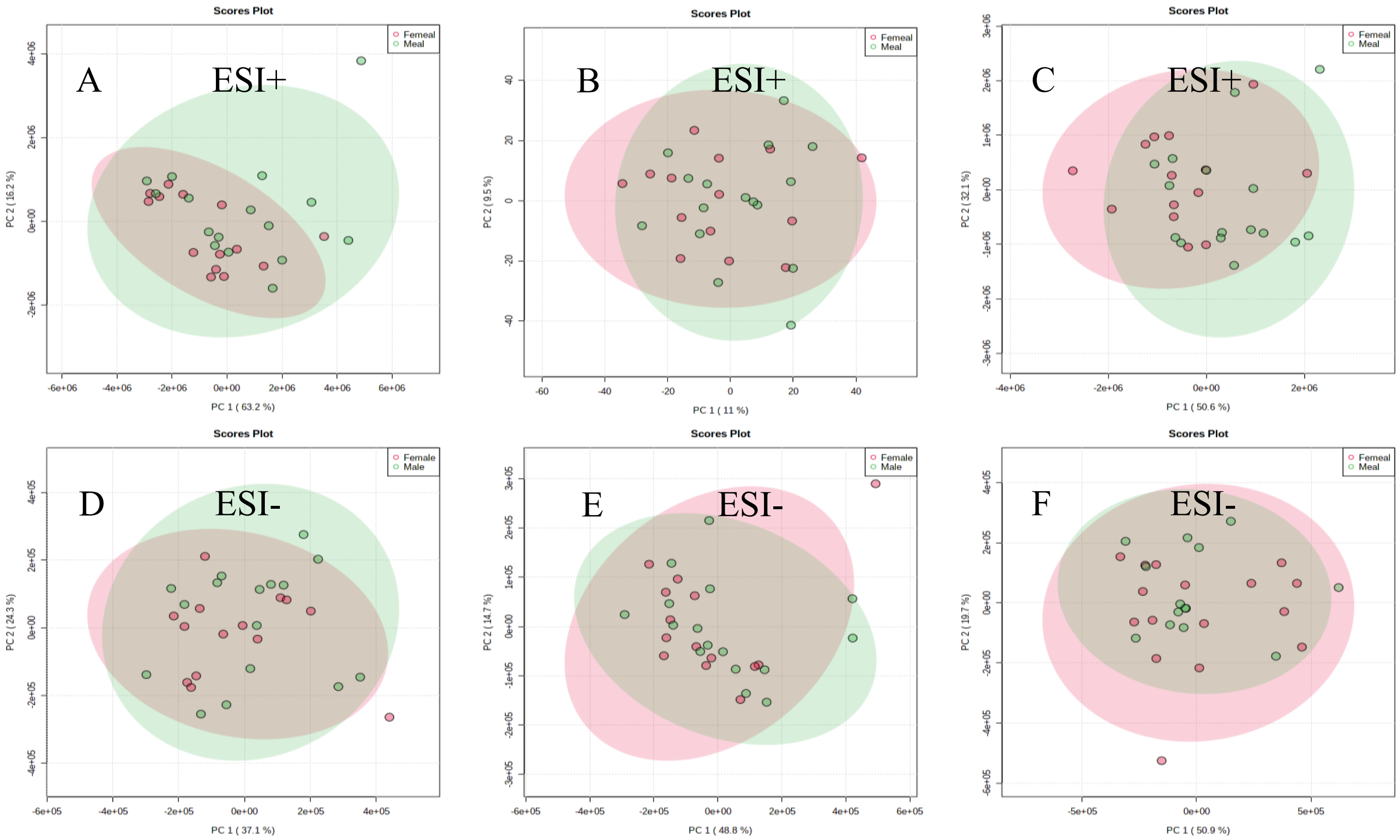

Supplementary Figure 2. The PCA score plots showing the comparison of the serum metabolites between female and male participants in the Control group **(A, D)**, Shi group **(B, E)**, and Xu **(C, F)** group in positive and negative ion modes.

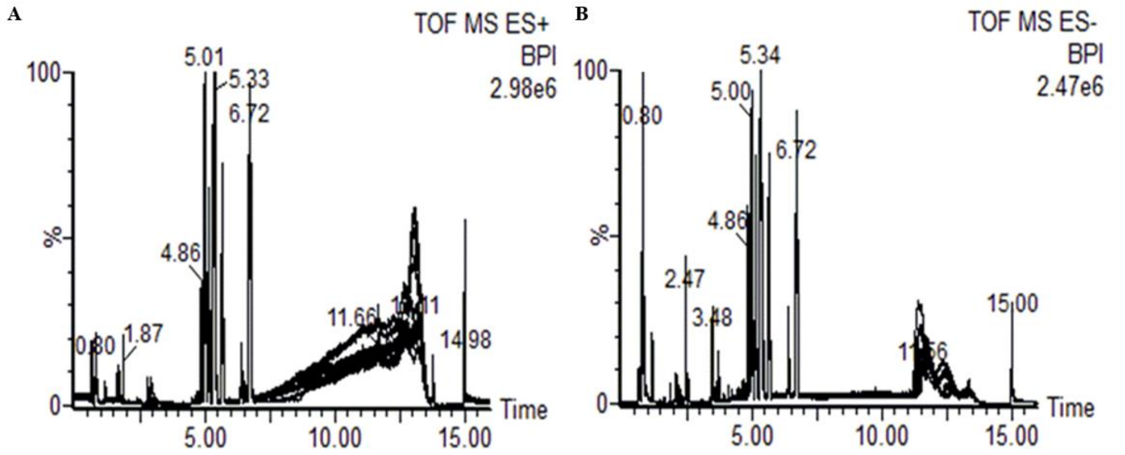

Supplementary Figure 3. The overlapped BPI chromatograms of all QC samples in the positive mode **(A)** and negative mode **(B)**

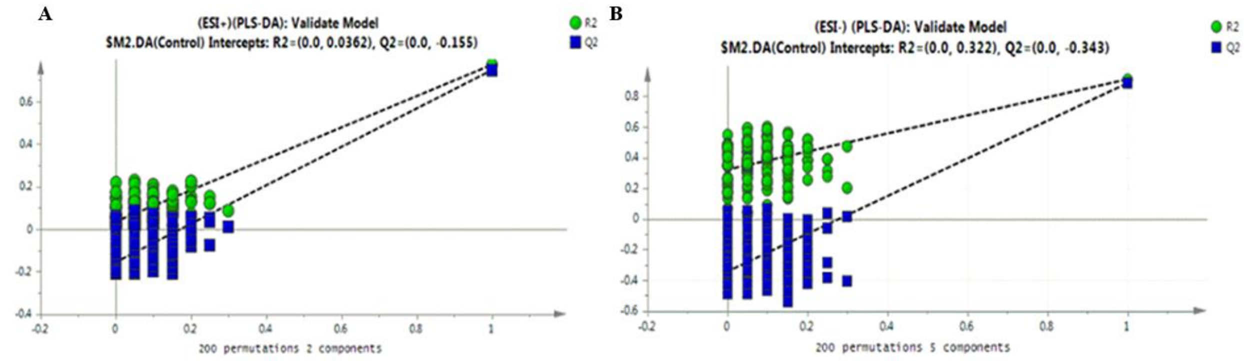

Supplementary Figure 4. Permutation test of the PLS-DA model in ESI positive mode **(A)** and in ESI negative mode **(B)**.

1.2 Supplementary Tables

Table S1. The detailed information of standards in this study

Compound	CAS number	Source
Glycine	56-40-6	Sigma-Aldrich (St. Louis, MO, USA)
β-Alanine	107-95-9	Sigma-Aldrich (St. Louis, MO, USA)
Sarcosine	107-97-1	Sigma-Aldrich (St. Louis, MO, USA)
Alanine	56-41-7	Sigma-Aldrich (St. Louis, MO, USA)
γ-Aminobutyric acid	56-12-2	Sigma-Aldrich (St. Louis, MO, USA)
L-2-Aminobutyric acid	1492-24-6	Sigma-Aldrich (St. Louis, MO, USA)
Serine	56-45-1	Sigma-Aldrich (St. Louis, MO, USA)
Proline	147-85-3	Sigma-Aldrich (St. Louis, MO, USA)
Valine	72-18-4	Sigma-Aldrich (St. Louis, MO, USA)
Threonine	72-19-5	Sigma-Aldrich (St. Louis, MO, USA)
Cysteine	52-90-4	Sigma-Aldrich (St. Louis, MO, USA)
Taurine	107-35-7	Sigma-Aldrich (St. Louis, MO, USA)
Pipecolinic acid	535-75-1	Sigma-Aldrich (St. Louis, MO, USA)
Leucine	61-90-5	Sigma-Aldrich (St. Louis, MO, USA)
Isoleucine	73-32-5	Sigma-Aldrich (St. Louis, MO, USA)
trans-4-Hydroxy-L-Proline	51-35-4	Sigma-Aldrich (St. Louis, MO, USA)
Asparagine	70-47-3	Sigma-Aldrich (St. Louis, MO, USA)
Ornithine	70-26-8	Sigma-Aldrich (St. Louis, MO, USA)

Asparagine acid	56-84-8	Sigma-Aldrich (St. Louis, MO, USA)
Glutamine	56-85-9	Tokyo Chemical Industry (Tokyo, Japan)
Lysine	56-87-1	Sigma-Aldrich (St. Louis, MO, USA)
Glutamic acid	56-86-0	Sigma-Aldrich (St. Louis, MO, USA)
Methionine	59-51-8	Sigma-Aldrich (St. Louis, MO, USA)
Histidine	71-00-1	Tokyo Chemical Industry (Tokyo, Japan)
L-2-Aminoadipic acid	542-32-5	Sigma-Aldrich (St. Louis, MO, USA)
Phenylalanine	63-91-2	Sigma-Aldrich (St. Louis, MO, USA)
1-Methyl-histidine	332-80-9	Sigma-Aldrich (St. Louis, MO, USA)
3-Methyl-histidine	368-16-1	Sigma-Aldrich (St. Louis, MO, USA)
Arginine	74-79-3	Sigma-Aldrich (St. Louis, MO, USA)
Citrulline	372-75-8	Sigma-Aldrich (St. Louis, MO, USA)
Tyrosine	60-18-4	Sigma-Aldrich (St. Louis, MO, USA)
Kynurenic acid	492-27-3	Sigma-Aldrich (St. Louis, MO, USA)
Tryptophan	73-22-3	Sigma-Aldrich (St. Louis, MO, USA)
Folic acid	59-30-3	Sigma-Aldrich (St. Louis, MO, USA)
L-Pyroglutamic acid	98-79-3	Sigma-Aldrich (St. Louis, MO, USA)
Hydroxyproline	51-35-4	Sigma-Aldrich (St. Louis, MO, USA)
Levocarnitine	541-15-1	Sigma-Aldrich (St. Louis, MO, USA)
Oleoylcarnitine	38677-66-6	Sigma-Aldrich (St. Louis, MO, USA)
Palmitoylcarnitine	1935-18-8	Sigma-Aldrich (St. Louis, MO, USA)
Sphingosine	123-78-4	Sigma-Aldrich (St. Louis, MO, USA)
Phytosphingosine	554-62-1	Sigma-Aldrich (St. Louis, MO, USA)
Stachydrine	471-87-4	Sigma-Aldrich (St. Louis, MO, USA)
Hypoxanthine	68-94-0	Sigma-Aldrich (St. Louis, MO, USA)
β-Pseudouridine	1445-07-4	Sigma-Aldrich (St. Louis, MO, USA)
Pantothenic acid	79-83-4	Sigma-Aldrich (St. Louis, MO, USA)
Indolelactic acid	1821-52-9	Sigma-Aldrich (St. Louis, MO, USA)
α-Hydroxyisocaproic acid	20312-37-2	Sigma-Aldrich (St. Louis, MO, USA)
Uric acid	69-93-2	Sigma-Aldrich (St. Louis, MO, USA)
Malic acid	6915-15-7	Sigma-Aldrich (St. Louis, MO, USA)
Nonanedioic acid	123-99-9	Sigma-Aldrich (St. Louis, MO, USA)
Citric acid	77-92-9	Sigma-Aldrich (St. Louis, MO, USA)

5-Hydroxyindoleacetic acid	54-16-0	Sigma-Aldrich (St. Louis, MO, USA)
Choline	62-49-7	Sigma-Aldrich (St. Louis, MO, USA)
Glycocholic acid	475-31-0	Sigma-Aldrich (St. Louis, MO, USA)
Taurocholic acid	81-24-3	Sigma-Aldrich (St. Louis, MO, USA)
Paraxanthine	611-59-6	Sigma-Aldrich (St. Louis, MO, USA)
Hippuric acid	495-69-2	Sigma-Aldrich (St. Louis, MO, USA)
3-Indoleacrylic acid	1204-06-4	Sigma-Aldrich (St. Louis, MO, USA)
Testosterone	58-22-0	Sigma-Aldrich (St. Louis, MO, USA)
Progesterone	57-83-0	Sigma-Aldrich (St. Louis, MO, USA)
Creatinine	60-27-5	Tokyo Chemical Industry (Tokyo, Japan)
Pentadecanoic acid	1002-84-2	Larodan (Stockholm Sweden)
Myristoleic acid	544-64-9	Larodan (Stockholm Sweden)
Linolenic acid	463-40-1	Larodan (Stockholm Sweden)
Arachidonic acid	506-32-1	Larodan (Stockholm Sweden)
Docosapentaenoic acid	24880-45-3	Larodan (Stockholm Sweden)
L-alanine-d_4	18806-29-6	Cambridge Isotope Laboratories (Andover, MA, USA)
L-tyrosine-$^{13}C_6$	201595-63-3	Cambridge Isotope Laboratories (Andover, MA, USA)
L-valine-d_8	35045-72-8	Cambridge Isotope Laboratories (Andover, MA, USA)
L-phenylalanine-$^{13}C_6$	180268-82-0	Cambridge Isotope Laboratories (Andover, MA, USA)
L-ornithine-d_2	3184-13-2	Cambridge Isotope Laboratories (Andover, MA, USA)
L-methionine-d_3	13010-53-2	Cambridge Isotope Laboratories (Andover, MA, USA)
L-leucine-d_3	87828-86-2	Cambridge Isotope Laboratories (Andover, MA, USA)
glycine-^{13}C,^{15}N	7299-33-4	Cambridge Isotope Laboratories (Andover, MA, USA)
DL-glutamatic acid-d_3	96927-56-9	Cambridge Isotope Laboratories (Andover, MA, USA)

Table S2. Case report form of CHD angina pectoris

Case selection criteria

SN	Inclusion Criteria	Yes	No	NA
**1**	Diagnostic criteria<br><br>① There has been no change in the frequency, duration, inducement, or remission pattern of angina pectoris in the past 60 days. There is no evidence of recent myocardial injury (serum myocardial markers troponin T or troponin I are normal, and the normal resting electrocardiogram showed ST segment significantly elevated).<br><br>② Coronary angiography or CTA examination within 1 year: the main coronary arteries (including the left anterior descending coronary artery, right coronary artery, left circumflex coronary artery, and left main coronary artery) stenosis lied in 50% -75%, or the collateral coronary arteries (except the main coronary arteries, such as diagonal branch, etc.) stenosis maintained in 50% -100%.	☐**1**	☐**0**	☐**99**
**2**	Age ≤75 years	□**1**	☐**0**	☐**99**
**3**	**TCM differentiation: Qi stagnation and blood stasis syndrome**; patient suffers Qi deficiency or QI-Yin deficiency;<br><br>TCM diagnostic criteria: **main symptoms + at least one minor symptom**<br><br>**Qi stagnation and blood stasis syndrome**<br><br>**Main symptoms:** chest tightness and pain (intermittent, scurrying pain, while the pain is mostly related to emotional changes)<br><br>**Minor symptom:** ① fullness chest and hypochondrium; ②sighing<br><br>**Qi deficiency and blood stasis syndrome:**<br><br>**Main symptoms:** chest tightness and dull pain, palpitation and shortness of breath, tiredness.<br><br>**Minor symptom:** Dizziness, insomnia and dreaminess.	☐**1**	☐**0**	☐**99**

**4**	Cardiac Function I-II (NYHA Cardiac Function Classification)	□**1**	☐**0**	☐**99**
**5**	Subjects informed and voluntarily signed informed consent	□**1**	☐**0**	☐**99**

Exclusion criteria

**SN**	**Exclusion Criteria (If the answer is "yes" to any of the following questions, the subject shall be excluded)**	**Yes**	**No**	**NA**
**1**	Renal insufficiency, male serum creatinine>2.5 mg/dl （>220umol/l） female>2.0 mg/dl（>175umol/l）	☐**1**	☐**0**	☐ **99**
**2**	Suffering from obvious liver disorders or ALT, AST indicators exceeding 3 times of the upper limit of normal value	☐**1**	☐**0**	☐ **99**
**3**	Patients with poor blood pressure control (systolic pressure> 160 mmHg, or diastolic pressure> 100 mmHg after taking antihypertensive drugs), At least 5 minutes of resting is needed before blood pressure measurement.	☐**1**	☐**0**	☐ **99**
**4**	Diabetics	□**1**	☐**0**	☐ **99**
**5**	Patients with severe chronic heart failure, severe arrhythmia, or a pacemaker, history of myocardial infarction	□**1**	☐**0**	☐ **99**
**6**	Patients with active pepticulcers and other hemorrhagic disorders	☐**1**	☐**0**	☐ **99**
**7**	Patients with malignant tumors, autoimmune disorders or hematological disorders	□**1**	☐**0**	☐ **99**
**8**	Patients with mental illness	□**1**	☐**0**	☐ **99**
**9**	Allergy to the component of test drug	□**1**	☐**0**	☐ **99**

**10**	Pregnancy or preparing pregnancy, breast-feeding	☐**1**	☐**0**	☐ **99**
**11**	The attendant has participated in or is participating in other clinical trials in the last three months	□**1**	☐**0**	☐ **99**

**Research number**	**Center number**	**Subject Initials**	**Subject number**	**Treatment date**	**Registration date**
|_|_|_|	|_|_|_|_|	|_|_|_|_|	|_|_|_|	**|_|_|day|_|_|month|_|_|_|_|year**	

Demographic information

**Date of birth**	|_|_|day|_|_|month|_|_|_|_|year	**Sex**	☐1 **male** ☐2 **female**
**Nationality**	□1 Han □2 Other→（Please specify： ）		
**Contact person**			
**Contact number**			
**Address**			

The basic characteristics of the subject

**Marital status**	☐1 Single ☐2 Married ☐4 Divorced ☐5 Widowed ☐6 Unknow
**Educational background**	□1 Illiteracy ☐2 Elementary school ☐3 Junior high school<br>☐4 High school / secondary school ☐5 Junior college<br>☐6 College and above ☐7 Unknow

**Nature of work**	□1Mental labor □2 Physical labor □88 Other→（Please specify： ）

Vital signs

**measurement item**	**measured value**	**unchecked**	**measuring position（if required）**
Height（cm）	|_|_|_|.|_|	□77	□99 NA
Weight（Kg）	|_|_|_|.|_|	□77	□99 NA
Respiration（times / minute）	|_|_|	□77	□99 NA
Resting heart rate（times / minute）	|_|_|_|	□77	□1Brachial artery □2 Carotid artery □3 Radial artery
Body temperature（℃）	|_|_|.|_|	□77	□4 Subaxillary □5 Ear
Systolic pressure（mmHg）	|_|_|_|	□77	□1Brachial artery □6 Ankle
Diastolic pressure（mmHg）	|_|_|_|	□77	□1Brachial artery □6 Ankle

Note: The vital signs and blood pressure need to be measured in a resting state, and the blood pressure is taken from the right upper arm blood pressure in the sitting position.

**Medical history** Does the subject have any other medical history / surgical history? □0 No □1 Yes→ Please fill in the following			
Name of disease or operation	Discovery date*	Disappearance date*	Whether to continue
	|_|_|day|_|_|month|_|_|_|_|year	|_|_|day|_|_|month|_|_|_|_|year	□1 Yes □0 NO

	|_|_|day|_|_|month|_|_|_|_|year	|_|_|day|_|_|month|_|_|_|_|year	☐1 Yes ☐0 NO
	|_|_|day|_|_|month|_|_|_|_|year	|_|_|day|_|_|month|_|_|_|_|year	☐1 Yes ☐0 NO
	|_|_|day|_|_|month|_|_|_|_|year	|_|_|day|_|_|month|_|_|_|_|year	☐1 Yes ☐0 NO
	|_|_|day|_|_|month|_|_|_|_|year	|_|_|day|_|_|month|_|_|_|_|year	☐1 Yes ☐0 NO

***Remarks: Please fill in the beginning and the ending date as much as possible. If the specific date is not clear, you can fill in UK.**

Pre-study medication Drug or non-drug therapy used within 3 months before this visit?□0 No □1 Yes→ Please fill in the form below

SN	Drug name	Single dose and unit	Frequency[2]	Route of administration[3]	Indications	Beginning date[4]	Ending date[4] or continuing
1						|_|_|day|_|_| month |_|_|_|_|year	|_|_|day|_|_|month |_|_|_|_|year or continuing ☐1
2						|_|_|day|_|_| month |_|_|_|_|year	|_|_|day|_|_|month |_|_|_|_|year or continuing ☐1
3						|_|_|day|_|_| month |_|_|_|_|year	|_|_|day|_|_|month |_|_|_|_|year or continuing ☐1
4						|_|_|day|_|_| month |_|_|_|_|year	|_|_|day|_|_|month |_|_|_|_|year or continuing ☐1

frontiers

5						|_|_|day|_|_| month<br><br>|_|_|_|_|year	|_|_|day|_|_|month<br><br>|_|_|_|_|year<br><br>or continuing □$_1$
6						|_|_|day|_|_| month<br><br>|_|_|_|_|year	|_|_|day|_|_|month<br><br>|_|_|_|_|year<br><br>or continuing □$_1$

Remarks:①Dose unit： mg/ug/Ml/g/IU/TABLET/CAPSULE/PUFF ②Frequency： BID、TID、QID、QOD、QM、PRN、UNKONWN、QD、others. ③Route of administration： oral、opical、subcutaneous、transdermal、intraocular、Intramuscular、respiratory/inhalation、intralesional、intraperitoneal、nasal、vaginal、rectal、sublingual、intravenous injection ④ Please fill in the beginning and the ending date as detail as possible. If the specific date is not clear, you can fill in UK.

Allergic history □$_0$ NO □$_1$Yes Description

Family history □$_0$ NO □$_1$Yes Description

Smoking history

Usage：<br><br>□$_1$ no smoking<br><br>□$_2$ occasional smoking, smoking □year<br><br>□$_3$ regular smoking, smoking □year<br><br>□$_4$ have smoked in the past, now quit smoking □year	Amount of smoking：	□$_1$ Less than 3sticks / day<br><br>□$_2$ 3-10 sticks / day<br><br>□$_3$ More than 10 sticks / day（Not CDISC control terminology）

**Beginning date：**	|_|_|day|_|_|month|_|_|_|_|year	Ending date：	|_|_|day|_|_|month|_|_|_|_| year
		Whether to continue：	□1Yes

<table>
<tr><td colspan="4">Alcohol drinking history</td></tr>
<tr><td colspan="2">Usage：
☐1 no drinking
☐2 occasional drinking, drinking ☐year
☐3 regular drinking, drinking ☐year
☐4 used to drink in the past, now quit drinking ☐year</td><td>Drinking amount：</td><td></td></tr>
<tr><td rowspan="2">Beginning date：</td><td rowspan="2">|_|_|day|_|_|month|_|_|_|_|year</td><td>Ending date：</td><td>|_|_|day|_|_|month|_|_|_|_|year</td></tr>
<tr><td>Whether to continue：</td><td>☐1Yes</td></tr>
</table>

General clinical information

Laboratory Examinations

<table>
<tr><td>laboratory indexes</td><td>Check value</td><td>Standard unit</td><td colspan="2">Clinical significance determination*</td></tr>
<tr><td colspan="3">Blood routine ampling date: |_|_|day|_|_|month|_|_|_|_|year</td><td>1 2 3 77</td><td>Exception description</td></tr>
<tr><td>(Red blood cell) RBC</td><td></td><td>$\times10^{12}$/L</td><td>☐ ☐ ☐ ☐</td><td></td></tr>
<tr><td>(Hemoglobin) HB</td><td></td><td>g/L</td><td>☐ ☐ ☐ ☐</td><td></td></tr>
<tr><td>(White blood cell) WBC</td><td></td><td>$\times10^{9}$/L</td><td>☐ ☐ ☐ ☐</td><td></td></tr>
<tr><td>Neutrophil %</td><td></td><td>%</td><td>☐ ☐ ☐ ☐</td><td></td></tr>
<tr><td>Platelet (PLT)</td><td></td><td>$\times10^{9}$/L</td><td>☐ ☐ ☐ ☐</td><td></td></tr>
<tr><td colspan="3">Urine routine Sampling date: |_|_|day|_|_|month|_|_|_|_|year</td><td>1 2 3 77</td><td>Exception description</td></tr>
</table>

Urine occult blood			□ □ □ □	
Urine WBC			□ □ □ □	
urine protein			□ □ □ □	
urine ketone			□ □ □ □	
urinary creatinine			□ □ □ □	
urine pregnant	□99 Unsuitable □77 Unchecked，reason ______________________<br>□0 Negative □1 Positive			
**Liver function Sampling date: |_|_|day|_|_|month|_|_|_|_|year**			**1 2 3 77**	**Exception description**
(Alanine transaminase) ALT		U/L	□ □ □ □	
(Aspartate aminotransferase) AST		U/L	□ □ □ □	
**Renal function Sampling date：|_|_|day|_|_|month|_|_|_|_|year**			**1 2 3 77**	**Exception description**
(Blood urea nitrogen) BUN		mmol/L	□ □ □ □	
(Uric acid) UA		umol/L	□ □ □ □	
(Serum creatinine) Scr		umol/L	□ □ □ □	
*** Clinical significance: 1 means normal; 2 means abnormal but no clinical significance; 3 means abnormal and clinical significance; 77 means unchecked.**				

Coronary angiography or CTA (Latest time)

Survey / measuring date：|_|_|day|_|_|month|_|_|_|_|year

Coronary revascularization	□1 Yes □0 NO
Complete coronary revascularization	□1 Yes □0 NO □77 Unknow
The way of revascularization	□1 PCI，total time，intervention time |_|_|day|_|_|month|_|_|_|_|year (last time)
	□2 CABG，total time，bypass grafting time |_|_|day|_|_|month|_|_|_|_|year (last time)
Involved coronary artery	□1 LM（the maximal stenosis degree： %）<br>□2 LAD（stenosis degree： %）<br>□3 LCX（the maximal stenosis degree： %）<br>□4 RCA（the maximal stenosis degree： %）<br>□5 PLA（the maximal stenosis degree： %）

TCM Syndrome Scale

The TCM diagnosis of CHD-associated syndromes was based upon the Standard Program for the Diagnosis and Treatment (2014) of Coronary Heart Disease with Angina Pectoris for Chinese Medicine Diagnosis and Treatment by the State Administration of TCM of China. As follows：

Shi syndrome

Main symptom	Scores	Criteria for symptom classification scores
Chest pain	0 3 6 9	Score 0: No. Score 3: The pain could be relieved after rest without affecting normal life; Score 6: It needs treatment with drugs and patient could go back to normal life after drugs. Score 9: The pain appears frequently that affects the normal life of patient (like dressing, eating, walking or even defecating could lead to the chest pain).

Chest tightness	0 3 6 9	Score 0: No. Score 3: Patient occasionally feels chest tightness that could be relived later. Score 6: It attacks the patient frequently but without affecting normal life and work. Score 9: The patient has to endure chest tightness constantly, which affects his/her normal life and work.
Minor symptom	Score	Criteria for symptom classification scores
Fullness chest and hypochondrium	0 2 4 6	Score 0: No. Score 2: Patient occasionally feels fullness chest and hypochondrium that could be self-relieved. Score 4: It attacks the patient frequently but without affecting normal life and work. Score 6: The patient has to endure fullness chest and hypochondrium constantly, which affects his/her normal life and work.
Sighing	0 2 4 6	Score 0: No. Score 2: Patient occasionally sighs that could be self-relieved. Score 4: It attacks the patient frequently but he could work. Score 6: The patient has to endure fullness chest and hypochondrium constantly, which affects his/her normal life and work.

Xu syndrome

main symptom	Score 0	Score 2	Score 4	Score 6
Chest pain	No	Patient suffers typical angina pectoris attack lasting several minutes every time and at least 2-3 times attack every week, or 1-3 times a day. Patient feels slight pain but sometimes has to take nitroglycerin	Patient suffers several typical angina pectoris attacks lasting several minutes to 10 minutes every time. Patient feels heavy pain and has to take nitroglycerin every time	Patient suffers several typical angina pectoris attacks every day leading to troubles of normal life or activities (like dressing coat or defecating and will last long time for every attack. Patient has to take nitroglycerin for many times

Chest tightness	No	Slight chest tightness	Patient feels apparent chest tightness and shows sighing breath, and short of breath after light activity	Patient feels stifling oppression in the chest and shows constant sighing.
Minor syndrome	Score 0	Score 1	Score 2	Score 3
Short of breath	No	Short of breath after common activities	Short of breath after light activities	Short of breath and dyspnea and tachypnea even without any activities
Tiredness and fatigue	No	Lassitude, weak strength, but could maintain normal life and activities	Mental fatigue, general weakness, and pushing himself to keep working	Suffering heavily mental fatigue and weakness, hard to maintain normal life
Spontaneous sweating	No	Slight sweating in normal life and worsening after light activities	Sweating in normal life and sweating after light activities	Sweating in normal life and sweating profusely after activities

Condition of tongue and pulse

Observation item	Specific signs
Tongue manifestation	□normal □enlarged □thin □tough □tender □light □purple □latent □red □ecchymosis □petechiae □teeth-marked □other：
Sublingual vein	□circuitous □engorged □purplish red □crimson purple □other：
Tongue coating	□ normal □thick □thin □lack □peeling □without □white □yellow □greasy □lack of fluid □ other：
Pulse manifestation	□ normal □deep □slow □rapid □thready □wiry □weak □slippery □unsmooth □irregular-rapid □irregularly intermittent

	☐regularly intermittent ☐ other：

Table S3. The detailed information of all detected lipids and fatty acids in the positive and negative mode.

RT (min)	Compounds	Precursor Ion	Product Ion	Cone(V)	Collision (V)
1.96	LPC (16:2)	492.3	184.1	10	30
2.06	LPC (14:0) *	468.3	184.1	10	30
2.22	LPC (18:4) *	516.4	184.1	10	30
2.30	LPC (20:5) *	542.3	184.1	10	30
2.38	LPC (16:1) *	494.3	184.1	10	30
2.51	LPC (15:0) *	482.3	184.1	10	30
2.67	LPE (18:2) *	478.3	337.3	30	20
2.71	LPC (18:3) *	518.3	184.1	10	30
2.75	LPE (20:4) *	502.3	361.3	30	20
2.76	LPC (22:6) *	568.3	184.1	10	30
2.78	LPC (20:4) *	544.3	184.1	10	30
2.84	LPC (17:1) *	508.3	184.1	10	30
2.95	LPE (16:0) *	454.3	313.3	30	20
2.99	LPC (16:0) *	496.3	184.1	10	30
2.99	LPE (20:5)	500.3	359.3	30	20
3.02	LPC (20:2)	548.4	184.1	10	30
3.12	LPC (20:3) *	546.4	184.1	10	30
3.15	FA14:1 *	225.2	225.2	10	10
3.25	LPC (22:5) *	570.4	184.1	10	30
3.30	LPE (18:1) *	480.3	339.3	30	20
3.31	LPC (18:2) *	520.3	184.1	10	30
3.37	FA 16:2	251.2	251.2	10	10
3.44	LPC (22:4) *	572.4	184.1	10	30
3.45	LPC (17:0) *	510.4	184.1	10	30
3.56	FA 20:5 *	301.2	301.2	10	10
3.57	FA 14:0 *	227.2	227.2	10	10
3.61	FA 18:3 *	277.2	277.2	10	10
3.81	FA 22:6 *	327.2	327.2	10	10

3.82	FA 16:1 *	253.2	253.2	10	10
3.90	LPC (18:0) *	524.4	184.1	10	30
3.90	LPC (18:1) *	522.4	184.1	10	30
3.99	FA 20:4 *	303.2	303.2	10	10
4.00	FA15:0 *	241.2	241.2	10	10
4.07	FA 18:2 *	279.2	279.2	10	10
4.09	LPC (20:1) *	550.4	184.1	10	30
4.23	FA 17:1 *	267.2	267.2	10	10
4.29	FA 22:5 *	329.2	329.2	10	10
4.34	FA 20:3 *	305.2	305.2	10	10
4.35	LPC (19:0)#	538.4	184.1	10	30
4.41	FA 16:0_d3#	258.3	258.3	10	10
4.42	FA 16:0 *	255.2	255.2	10	10
4.64	FA 22:4 *	331.3	331.3	10	10
4.65	FA 18:1 *	281.2	281.2	10	10
4.82	LPC (20:0) *	552.4	184.1	10	30
4.83	FA 20:2 *	307.3	307.3	10	10
4.87	FA 17:0 *	269.2	269.2	10	10
4.93	LPC (22:1) *	578.4	184.1	10	30
5.28	LPC (21:0)	566.4	184.1	10	30
5.34	FA 18:0_d3#	286.3	286.3	10	10
5.35	FA 18:0 *	283.3	283.3	10	10
5.80	LPC (22:0) *	580.4	184.1	10	30
5.85	FA 19:0#	297.2	297.2	10	10
5.85	LPC (24:1) *	606.5	184.1	10	30
6.28	PE(12:0/13:0)#	594.4	453.4	31	50
6.33	FA 20:0 *	311.3	311.3	10	10
6.43	FA 22:1 *	337.3	337.3	10	10
6.48	SM(d18:1/12:0)#	647.5	184.1	50	30
6.61	SM(d16:1/16:1) *	673.5	184.1	50	30
6.80	LPC (24:0) *	608.5	184.1	10	30
7.07	SM(d18:1/15:1) *	687.5	184.1	50	30
7.12	PC(18:3/16:2) *	752.5	184.1	40	15
7.36	SM(d16:0/16:1) *	675.5	184.1	50	30
7.37	FA 22:0 *	339.3	339.3	10	10
7.38	PC(12:0/16:0) *	678.5	184.1	40	15
7.47	PC(15:0/16:0) *	720.6	184.1	40	15
7.51	SM(d16:0/18:2) *	701.6	184.1	50	30
7.67	PC(20:4/22:6) *	854.6	184.1	40	15
7.70	PC(18:2/20:5) *	804.6	184.1	40	15
7.71	PC(16:1/18:3) *	754.5	184.1	40	15
7.78	SM(d16:0/17:0) *	691.6	184.1	50	30
7.79	SM(d16:0/20:3) *	727.6	184.1	50	30
7.80	PC(10:0/19:1)	690.5	184.1	40	15
7.80	SM(d16:0/17:1) *	689.6	184.1	50	30

7.85	PC(15:0/22:6)*	792.6	184.1	40	15
7.90	PC(20:3/22:6) *	856.6	184.1	40	15
7.93	PC(16:0/20:5) *	780.6	184.1	40	15
7.96	SM(d18:1/17:1) *	715.6	184.1	50	30
8.02	SM(d20:0/22:6)	805.6	184.1	50	30
8.13	PC(15:0/18:2) *	744.6	184.1	40	15
8.17	PC(16:0/18:3) *	756.6	184.1	40	15
8.17	PC(16:1/18:2) *	756.6	184.1	40	15
8.21	PC(14:0/16:1) *	704.5	184.1	40	15
8.21	SM(d16:0/18:1) *	703.6	184.1	50	30
8.22	PE(16:1 /18:2) *	714.5	573.5	31	50
8.25	SM(d20:0/22:5)	807.6	184.1	50	30
8.26	PE(16:0 /20:4) *	740.5	599.5	31	50
8.28	PC(17:1/18:2) *	770.6	184.1	40	15
8.37	PC(16:0/16:1) *	732.6	184.1	40	15
8.37	PC(16:1/16:1) *	730.5	184.1	40	15
8.39	PC(18:1/22:6) *	832.6	184.1	40	15
8.39	PC(18:2/22:6)	830.6	184.1	40	15
8.40	SM(d16:0/20:2) *	729.6	184.1	50	30
8.42	PE(16:0 /22:6) *	764.5	623.5	31	50
8.47	PC(20:2/22:6)	858.6	184.1	40	15
8.49	SM(d16:0/22:3) *	755.6	184.1	50	30
8.50	SM(d16:0/18:0) *	705.6	184.1	50	30
8.57	PC(14:0/22:6) *	778.5	184.1	40	15
8.57	PC(16:0/17:1) *	746.6	184.1	40	15
8.63	PC(18:0/20:5) *	808.6	184.1	40	15
8.64	SM(d16:0/19:1) *	717.6	184.1	50	30
8.71	PE(16:1 /18:1) *	716.5	575.5	31	50
8.81	PE(16:0 /22:5) *	766.5	625.5	31	50
8.87	PC(17:0/20:4) *	796.6	184.1	40	15
8.92	PC(16:1/19:1) *	772.6	184.1	40	15
8.94	PC(16:0/16:0) *	734.6	184.1	40	15
8.95	PC(15:1/18:2) *	742.5	184.1	40	15
8.96	SM(d18:1/19:1) *	743.6	184.1	50	30
9.00	PC(18:0/22:6) *	834.6	184.1	40	15
9.04	SM(d16:0/20:1) *	731.6	184.1	50	30
9.05	SM(d18:2/17:1) *	713.6	184.1	50	30
9.08	PC(20:1/22:6) *	860.6	184.1	40	15
9.11	Cer(d18:1/16:0) *	538.5	264.3	20	30
9.11	PC(18:2/19:1) *	798.6	184.1	40	15
9.19	PC(18:2/22:3) *	836.6	184.1	40	15
9.20	PE(18:0 /22:5) *	794.6	653.6	31	50
9.25	SM(d20:0/22:4)	809.7	184.1	50	30

9.40	SM(d16:0/19:0)	719.6	184.1	50	30
9.43	PC(20:0/22:6) *	862.6	184.1	40	15
9.47	PC(16:0/19:1) *	774.6	184.1	40	15
9.50	Cer(d18:1/17:0)#	552.5	264.3	20	30
9.53	SM(d18:1/19:0) *	745.6	184.1	50	30
9.63	PC(18:2/19:0) *	800.6	184.1	40	15
9.66	PC(16:0/18:0) *	762.6	184.1	40	15
9.68	PC(18:0/22:4) *	838.6	184.1	40	15
9.81	PC(18:0/18:1) *	788.6	184.1	40	15
9.86	PC(18:2/20:0) *	814.6	184.1	40	15
9.87	Cer(d18:1/18:0) *	566.6	264.3	20	30
9.96	PC 42:5-2	864.6	184.1	40	15
9.98	PC(16:2/24:1)	840.6	184.1	40	15
9.99	PE(16:0 /18:1) *	718.5	577.5	31	50
10.16	PC(20:0/22:4) *	866.7	184.1	40	15
10.22	SM(d18:1/25:0)	829.7	184.1	50	30
10.22	SM(d20:0/19:1) *	773.7	184.1	50	30
10.32	PC(16:0/20:0) *	790.6	184.1	40	15
10.37	PC(16:0/22:1) *	816.6	184.1	40	15
10.47	PC(20:5/23:0)	878.7	184.1	40	15
10.49	SM(d18:1/24:1) *	813.7	184.1	50	30
10.57	Cer(d18:1/20:0) *	594.6	264.3	20	30
10.62	PC(16:2/26:1)	868.7	184.1	40	15
10.71	Cer(d18:2/24:1) *	646.6	262.2	20	30
10.85	SM(d16:1/25:0)	801.7	184.1	50	30
10.91	PC(19:0/19:0)#	818.6	184.1	40	15
11.01	Cer(d18:2/22:0) *	620.6	262.2	20	30
11.05	SM(d18:2/25:0)	827.7	184.1	50	30
11.17	SM(d16:0/26:1) *	815.7	184.1	50	30
11.18	Cer(d18:1/24:1) *	648.6	264.3	20	30
11.21	Cer(d18:1/22:0) *	622.6	264.3	20	30
11.26	PC(18:0/24:2) *	870.7	184.1	40	15
11.50	Cer(d18:1/22:2) *	618.6	264.3	20	30
11.79	Cer(d18:1/24:0) *	650.6	264.3	20	30
12.04	Cer(d18:1/25:0)	664.7	264.3	20	30
12.12	Cer(d18:1/26:1) *	676.7	264.3	20	30
12.54	TG(18:4/16:0/18:2) *	868.6	597.4	35	20
12.77	TG(16:0/14:0/18:3) *	818.7	547.4	35	20
12.83	TG(16:0/16:1/18:3)	844.6	545.4	35	20
12.89	TG(16:0/18:2/18:3)	870.6	597.4	35	20
12.94	TG(18:1/18:2/18:3)	896.6	625.4	35	20
13.11	TG(16:1/14:0/18:1)	820.7	549.4	35	20
13.16	TG(16:0/16:2/18:1) *	846.6	575.4	35	20
13.18	TG(15:0/15:0/15:0)#	782.55	523.4	35	20
13.21	TG(16:0/18:2/18:2)	872.6	573.4	35	20

13.24	TG(18:1/18:2/18:2)	898.6	627.4	35	20
13.35	TG(16:0/14:0/18:1)	822.6	549.4	35	20
13.36	TG(16:0/16:1/18:1)	848.6	575.4	35	20
13.39	TG(16:0/16:0/20:3) *	874.6	603.4	35	20
13.41	TG(16:0/18:1/20:3) *	900.8	629.4	35	20
13.46	TG(18:0/14:0/16:0) *	824.6	551.4	35	20
13.47	TG(16:0/16:0/18:1)	850.6	577.4	35	20
13.49	TG(18:0/16:0/18:2) *	876.6	575.4	35	20
13.50	TG(18:0/18:1/18:2)	902.6	629.4	35	20
13.54	TG(18:0/16:0/16:0)	852.6	579.4	35	20
13.55	TG(18:0/18:1/18:1)	904.8	631.4	35	20
13.60	TG(18:0/18:0/18:1)	906.6	605.4	35	20

* indentified with represented standards; # internal standards

Table S4. Repeatability of the 4 internal standards for untargeted metabolomics in serum in positive and negative mode

RT(min)	Compound	RSD%(ESI+)	RSD%(ESI-)
1.64	Phenylalanine-d5	12.46	10.68
7.67	LPC 19:0	15.11	12.54
10.9	SM(d18:0/12:0)	9.25	10.43
11.84	PE(12:0/13:0)	5.57	5.50

Table S5. Repeatability of the 11 Internal Standards of the target amino acids in serum

RT(min)	Compound	RSD%
2.71	Leucine (D3)	3.55
2.76	Phenylalanine (13C6)	2.91
3.39	Methionine (D3)	4.47
3.61	Valine (D8)	1.63
3.98	Tyrosine (13C6)	4.99
5.09	Alanine (D4)	3.77
6.02	Glycine (13C; 15N)	12.57
6.08	Glutamate (D3)	8.16
7.87	Citrulline (D2)	11.74
9.96	Arginine (13C; D4)	15.21
10.77	Ornithine (D2)	15.41

Table S6. Repeatability of the 9 internal standards for targeted lipids and fatty acids in serum

RT(min)	Compound	RSD%
4.35	LPC 19:0	6.41
4.41	Hexadecanoic -16,16,16-D3 Acid	13.33
5.34	Stearic acid-18,18,18-d3	13.57
5.85	Nonadecanoic acid	13.25
6.28	PE(12:0/13:0)	10.81
6.48	SM(d18:0/12:0)	6.68
9.5	Ceramide(d18:1/17:0)	9.96
10.91	PC(19:0/19:0)	7.63
13.18	TG (15:0/15:0/15:0)	7.83

Table S7. Identification of potential biomarkers in serum based on untargeted metabolomics

Rt (min)	Ion mode	Assigned identity	Molecularformula	Theoretical extract mass (Da)	Mean measured mass (Da)	Mass Accuracy (ppm)	Product ion of ESI/MS2
0.70	ESI (+)	Arginine	$C_6H_{14}N_4O_2$	175.1195	175.1198	1.7	112.0895, 70.0650
0.70	ESI (+)	Histidine	$C_6H_9N_3O_2$	156.0773	156.0775	1.3	110.0718, 83.0619
0.77	ESI (+)	Choline	$C_5H_{14}NO^+$	104.1075	104.1078	2.9	60.0804
0.77	ESI (+)	Carnitine	$C_7H_{15}NO_3$	162.113	162.1136	3.7	103.0398, 85.0294
0.79	ESI (+)	Creatinine	$C_4H_7N_3O$	114.0667	114.0669	1.8	86.0776
0.82	ESI (+)	Proline	$C_5H_9NO_2$	116.0712	116.0712	0.0	70.0658
0.83	ESI (+)	Stachydrine	$C_7H_{13}NO_2$	144.1025	144.1028	2.1	58.0650
0.87	ESI (-)	Uric acid	$C_5H_4N_4O_3$	167.0205	167.022	9.0	124.0139, 83.0123
0.88	ESI (-)	Malic acid	$C_4H_6O_5$	133.0137	133.0141	3.0	115.0028, 71.0146
1.12	ESI (-)	β-Pseudouridine	$C_9H_{12}N_2O_6$	243.0617	243.0633	6.6	153.0311, 110.0261
1.12	ESI (-)	Citric acid	$C_6H_8O_7$	191.0192	191.0201	4.7	110.958, 87.059
1.16	ESI (-)	L-Pyroglutamic acid	$C_5H_7NO_3$	128.0348	128.0347	-0.8	82.0286
1.17	ESI (+)	Hypoxanthine	$C_5H_4N_4O$	137.0463	137.0466	2.2	110.0357, 55.0288
1.20	ESI (+)	Adenosine	$C_{10}H_{13}N_5O_4$	268.1046	268.104	-2.2	212.0930, 136.0766
1.21	ESI (+)	Tyrosine	$C_9H_{11}NO_3$	182.0817	182.0819	1.1	136.0766, 91.0549

1.65	ESI (+)	Phenylalanine	$C_9H_{11}NO_2$	166.0868	166.0872	2.4	120.0827, 103.0530
1.69	ESI (+)	Theobromine	$C_7H_8N_4O_2$	181.0726	181.0727	0.6	138.0654, 89.0748
1.70	ESI (+)	Pantothenic acid	$C_9H_{17}NO_5$	220.1185	220.1182	-1.4	90.0554
1.79	ESI (+)	Folic acid	$C_{19}H_{19}N_7O_6$	442.1475	442.1491	3.6	295.0936
1.83	ESI (+)	Paraxanthine	$C_7H_8N_4O_2$	181.0726	181.0728	1.1	121.0283
1.84	ESI (-)	Tryptophan	$C_{11}H_{12}N_2O_2$	203.0821	203.0832	4.4	116.0504
2.13	ESI (-)	5-Hydroxyindoleacetic acid	$C_{10}H_9NO_3$	190.0504	190.0505	0.5	160.0393, 146.0599
2.29	ESI (+)	Hippuric acid	$C_9H_9NO_3$	180.0661	180.0662	0.6	150.0338, 77.0385
2.5	ESI (-)	α-Hydroxyisocaproic acid	$C_6H_{12}O_3$	131.0708	131.0717	6.9	85.0667, 69.0359
2.69	ESI (-)	Indolelactic acid	$C_{11}H_{11}NO_3$	204.0661	204.0657	-2.0	158.0603, 168.055
2.69	ESI (+)	3-Indoleacrylicacid	$C_{11}H_9NO_2$	188.0712	188.0709	-1.6	170.0600, 152.0495
2.8	ESI (-)	Nonanedioic acid	$C_9H_{16}O_4$	187.097	187.0979	4.8	125.0959, 97.0645
3.04	ESI (+)	Octanoylcarnitine	$C_{15}H_{29}NO_4$	288.2175	288.2178	1.0	229.1430, 85.0277
3.23	ESI (-)	Taurocholic acid	$C_{26}H_{45}NO_7S$	514.2838	514.2831	-1.4	512.6697, 293.6768
3.42	ESI (-)	Glycochenodeoxycholic acid	$C_{26}H_{43}NO_5$	448.3063	448.3067	0.9	386.3074, 74.023
3.57	ESI (+)	Glycocholic acid	$C_{26}H_{43}NO_6$	466.3169	466.317	0.2	412.2845, 430.2950
4.09	ESI (-)	Cholic Acid	$C_{24}H_{40}O_5$	407.2797	407.2791	-1.5	343.2645, 289.2174
4.11	ESI (+)	Testosterone	$C_{19}H_{28}O_2$	289.2168	289.2173	1.7	97.0651, 109.0634
4.17	ESI (+)	Phytosphingosine	$C_{18}H_{39}NO_3$	318.3008	318.3012	1.3	282.2793, 60.0452

4.32	ESI (+)	Sphingosine	$C_{18}H_{37}NO_2$	300.2903	300.2897	-2.0	282.2787, 211.2050
4.48	ESI (-)	LPE (14:0)	$C_{19}H_{40}NO_7P$	424.2462	424.248	3.8	227.2017, 209.1911
4.51	ESI (+)	LPC (14:0)	$C_{22}H_{46}NO_7P$	468.309	468.3091	0.2	184.0718
4.87	ESI (+)	Palmitoylcarnitine	$C_{23}H_{45}NO_4$	400.3427	400.3424	-0.7	341.2687, 85.0290
4.87	ESI (-)	LPC (15:0)	$C_{23}H_{48}NO_7P$	526.3145	526.3142	-0.6	241.2165
5.05	ESI (+)	Oleoylcarnitine	$C_{25}H_{47}NO_4$	426.3583	426.358	-0.7	85.0280, 367.2847
5.30	ESI (-)	LPE (16:0)	$C_{21}H_{44}NO_7P$	452.2777	452.2784	1.5	255.2330, 237.2224
5.32	ESI (+)	Progesterone	$C_{21}H_{30}O_2$	315.2324	315.2339	4.8	283.1074, 97.0653
5.34	ESI (-)	LPC (16:0)	$C_{24}H_{51}NO_7P$	540.3301	540.3314	2.4	255.2337
5.42	ESI (-)	LPE (18:1)	$C_{23}H_{46}NO_7P$	478.294	478.2934	1.3	281.2486, 263.2380
6.65	ESI (-)	LPE (18:0)	$C_{23}H_{48}NO_7P$	480.309	480.309	0.0	283.2643, 265.2537
6.72	ESI (-)	LPC (18:0)	$C_{26}H_{54}NO_7P$	568.3614	568.3612	-0.4	283.2635
8.31	ESI (-)	Linolenic acid	$C_{18}H_{30}O_2$	277.2168	277.218	4.3	233.2223, 164.9045
8.79	ESI (+)	LPC (20:0)	$C_{28}H_{58}NO_7P$	552.4029	552.4025	-0.7	184.0718
9.50	ESI (+)	Arachidonic acid	$C_{20}H_{32}O_2$	305.2481	305.2481	0.0	259.2038
11.5	ESI (+)	PC (22:0/0:0)	$C_{30}H_{62}NO_7P$	580.4342	580.4335	-1.2	184.0718

Table S8. The pathway of the metabolites between the Control group and Shi group

Pathway Name	Metabolites
Aminoacyl-tRNA biosynthesis	Histidine; Glutamine; Cysteine
Purine metabolism	Glutamine; Hypoxanthine; Uric acid
Alanine, aspartate and glutamate metabolism	Glutamine; Citric acid
Glyoxylate and dicarboxylate metabolism	Glutamine; Citric acid
Biosynthesis of unsaturated fatty acids	Arachidonic acid; Linolenic acid
D-Glutamine and D-glutamate metabolism	Glutamine
Nitrogen metabolism	Glutamine
Thiamine metabolism	Cysteine
Taurine and hypotaurine metabolism	Cysteine
alpha-Linolenic acid metabolism	Linolenic acid
Arginine biosynthesis	Glutamine
Histidine metabolism	Histidine
Pantothenate and CoA biosynthesis	Cysteine
Tricarboxylic acid cycle (TCA cycle)	Citric acid
beta-Alanine metabolism	Histidine
Glutathione metabolism	Cysteine
Glycine, serine and threonine metabolism	Cysteine
Cysteine and methionine metabolism	Cysteine
Glycerophospholipid metabolism	LPC (20:2)
Arachidonic acid metabolism	Arachidonic acid
Pyrimidine metabolism	Glutamine

Table S9. The pathway of the metabolites between the Control group and Xu group

Pathway Name	Metabolites
Taurine and hypotaurine metabolism	Cysteine; Taurocholic acid
Aminoacyl-tRNA biosynthesis	Histidine; Cysteine
Sphingolipid metabolism	Cer(d18:1/20:0); Cer(d18:2/22:0); Cer(d18:1/25:0); SM(d20:0/22:6)
Purine metabolism	Glutamine; Hypoxanthine; Uric acid
Alanine, aspartate and glutamate metabolism	Glutamine; Citric acid
Glyoxylate and dicarboxylate metabolism	Citric acid; Glutamine
D-Glutamine and D-glutamate metabolism	Glutamine
Nitrogen metabolism	Glutamine
Primary bile acid biosynthesis	Glycocholic acid; Taurocholic acid
Thiamine metabolism	Cysteine
Arginine biosynthesis	Glutamine
Histidine metabolism	Histidine
Pantothenate and CoA biosynthesis	Cysteine
Tricarboxylic acid cycle (TCA cycle)	Citric acid
beta-Alanine metabolism	Histidine
Glutathione metabolism	Cysteine
Glycine, serine and threonine metabolism	Cysteine
Cysteine and methionine metabolism	Cysteine
Biosynthesis of unsaturated fatty acids	Arachidonic acid
Glycerophospholipid metabolism	LPC (20:2)

Arachidonic acid metabolism	Arachidonic acid
Pyrimidine metabolism	Glutamine

Table S10. Metabolite differences between Xu and Shi subtype CHD patients.

Subtype	Name	Change	Taxonomy Class	General Role	Biological role	Kegg Pathway	Health condition associations (Human Metabolomics Data Base)	Chemical disease associations (PubChem)
Xu	Cer(d18:1/20:0)	# ↓	Sphingolipids	Membrane stabilizer<br>Energy source<br>Energy storage<br>Inflammatory<br>Insulin signaling antagonist<br>Second messenger<br>Signaling molecule	important cellular signals for inducing apoptosis		metabolic syndrome, lipotoxicity	Acne Vulgaris, Acute Coronary Syndrome, Albuminuria, Anemia, Sickle Cell, Angina, Stable, Cardiovascular Diseases, Constriction, Pathologic, Coronary Artery Disease, Cyanosis, Diabetes Mellitus, Diabetes Mellitus, Type 2, Dyslipidemias, Fatty Liver, Heart Defects, Congenital, Hypertension, Hypoglycemia, Hypoxia, Inflammation, Insulin Resistance, Metabolic Syndrome, Myocardial Infarction, Myocardial Ischemia, Plaque, Amyloid, Polycythemia, Renal Insufficiency, Chronic
	Cer(d18:1/25:0)	# ↓	Sphingolipids		important cellular signals for inducing apoptosis		metabolic syndrome, lipotoxicity	Colorectal cancer
	Cer(d18:2/22:0)	# ↓	Sphingolipids					
	Glycocholic acid	# ↑	Steroids and steroid derivatives	Membrane stabilizer<br>Energy source<br>Energy storage<br>Signaling molecule	uptake of vitamins & fats	1 27-Hydroxylase Deficiency<br>2 Bile Acid Biosynthesis<br>3 Cerebrotendinous Xanthomatosis (CTX)<br>4 Congenital Bile Acid Synthesis Defect Type II<br>5 Congenital Bile Acid Synthesis Defect Type III<br>6 Familial Hypercholanemia (FHCA)<br>7 Zellweger Syndrome	Acute liver failure, Intrahepatic biliary hypoplasia, Portal vein obstruction, Alpha-1-antitrypsin deficiency, Choledochal cyst, Galactosemia type 1, Celiac disease, Wilson's disease, Cystic fibrosis, Neonatal hepatitis, Chronic active hepatitis, Metabolism and nutrition disorders: Glycogen storage disease, Hepatobiliary disorders: Hepatobiliary disease, Biliary atresia	Chemical and Drug Induced Liver Injury
	Taurocholic acid	# ↑	Steroids and steroid derivatives	Membrane stabilizer<br>Energy source<br>Energy storage<br>Hormone<br>Signaling molecule	uptake of vitamins & fats	1 27-Hydroxylase Deficiency<br>2 Bile Acid Biosynthesis<br>3 Cerebrotendinous Xanthomatosis (CTX)<br>4 Congenital Bile Acid Synthesis Defect Type II<br>5 Congenital Bile Acid Synthesis Defect Type III	Biliary atresia	Chemical and Drug Induced Liver Injury, Cholestasis, Disease Models, Animal, Edema, Hemorrhage, Hyperemia, Leiomyosarcoma, Lung Injury, Necrosis, Neoplasm Invasiveness, Pancreatitis, Stomach Diseases, Stomach Ulcer

						6 Familial Hypercholanemia (FHCA)<br>7 Zellweger Syndrome		
	Linolenic acid	# ↓	Fatty Acyls	Essential fatty acid<br>Membrane stabilizer<br>Energy source<br>Energy storage<br>Nutrient	fatty acid metabolism & inflammation	Alpha Linolenic Acid and Linoleic Acid Metabolism	Essential hypertension, Vascular disorders: Hypertension, Cancer: Thyroid cancer	Brain Injuries, Chemical and Drug Induced Liver Injury, Depressive Disorder, Disease Models, Animal, Drug-Related Side Effects and Adverse Reactions, Inflammation, Intracranial Arteriosclerosis, Memory Disorders, Movement Disorders, Nervous System Diseases. Organophosphate Poisoning, Tobacco Use Disorder, Vascular Diseases
**Shi**	Citric acid	# ↓	Carboxylic acids and derivatives		mitochondrial function	1 2-ketoglutarate dehydrogenase complex deficiency<br>2 Citric Acid Cycle<br>3 Congenital lactic acidosis<br>4 Fumarase deficiency<br>5 Glutaminolysis and Cancer<br>6 Mitochondrial complex II deficiency<br>7 Pyruvate dehydrogenase deficiency (E2)<br>8 Pyruvate dehydrogenase deficiency (E3)<br>9 The oncogenic action of 2-hydroxyglutarate<br>10 The oncogenic action of D-2-hydroxyglutarate in Hydroxygluaricaciduria<br>11 The Oncogenic Action of Fumarate<br>12 The oncogenic action of L-2-hydroxyglutarate in Hydroxygluaricaciduria<br>13 The Oncogenic Action of Succinate<br>14 Transfer of Acetyl Groups into Mitochondria<br>15 Warburg Effect	Schizophrenia, Paraquat poisoning, Hyperoxalemia, Rhabdomyolysis, Cancer: Lung cancer	Arthritis, Experimental, Cattle Diseases, Chromosome Breakage, Cough, Endometritis, Kidney Calculi, Myocardial Ischemia, Puerperal Infection, Ureteral Calculi, Urinary Bladder, Overactive, Vascular Diseases, Acidosis, Acidosis, Renal Tubular, Extravasation of Diagnostic and Therapeutic Materials, Hypocalcemia, Nephrocalcinosis, Papilloma, Precancerous Conditions, Sjogren's Syndrome, Urinary Bladder Neoplasms, Urinary Calculi, Ventricular Fibrillation
	LPC (20:2)	# ↑	Glycerophospholipids	Energy source<br>Membrane stabilizer<br>Energy storage	catalyzes the transfer of the fatty acids of position sn-2 of phosphatidylcholine to the free cholesterol in plasma			Coronary Disease, Atherosclerosis
